# Cancer progression by breast tumors with Pit-1-overexpression is blocked by inhibition of metalloproteinase (MMP)-13

**DOI:** 10.1186/s13058-014-0505-8

**Published:** 2014-12-20

**Authors:** Juan Sendon-Lago, Samuel Seoane, Noemi Eiro, Maria A Bermudez, Manuel Macia, Tomas Garcia-Caballero, Francisco J Vizoso, Roman Perez-Fernandez

**Affiliations:** 1 Department of Physiology- Center for Research in Molecular Medicine and Chronic Diseases (CIMUS), School of Medicine, University of Santiago de Compostela, Praza do Obradoiro, Santiago de Compostela, 15782 Spain; 2 Unidad de Investigación, Fundacion Hospital de Jove, Avenida Eduardo Castro, Gijón, 33290 Spain; 3 Departments of Obstetrics and Gynecology, School of Medicine, University of Santiago de Compostela, Praza do Obradoiro, Santiago de Compostela, 15782 Spain; 4 Departments of Morphological Sciences, School of Medicine, University of Santiago de Compostela, Praza do Obradoiro, Santiago de Compostela, 15782 Spain

## Abstract

**Introduction:**

The POU class 1 homeobox 1 transcription factor (POU1F1, also known as Pit-1) is expressed in the mammary gland and its overexpression induces profound phenotypic changes in proteins involved in cell proliferation, apoptosis, and invasion. Patients with breast cancer and elevated expression of Pit-1 show a positive correlation with the occurrence of distant metastasis. In this study we evaluate the relationship between Pit-1 and two collagenases: matrix metalloproteinase-1 (MMP-1) and matrix metalloproteinase-13 (MMP-13), which have been related to metastasis in breast cancer.

**Methods:**

We began by transfecting the MCF-7 and MDA-MB-231 human breast adenocarcinoma cell lines with the Pit-1 overexpression vector (pRSV-hPit-1). Afterward, the mRNA, protein, and transcriptional regulation of both MMP-1 and MMP-13 were evaluated by real-time PCR, Western blot, chromatin immunoprecipitation (ChIP), and luciferase reporter assays. We also evaluated Pit-1 overexpression with MMP-1 and MMP-13 knockdown in a severe combined immunodeficiency (SCID) mouse tumor xenograft model. Finally, by immunohistochemistry we correlated Pit-1 with MMP-1 and MMP-13 protein expression in 110 human breast tumors samples.

**Results:**

Our data show that Pit-1 increases mRNA and protein of both MMP-1 and MMP-13 through direct transcriptional regulation. In SCID mice, knockdown of MMP-13 completely blocked lung metastasis in Pit-1-overexpressing MCF-7 cells injected into the mammary fat pad. In breast cancer patients, expression of Pit-1 was found to be positively correlated with the presence of both MMP-1 and MMP-13.

**Conclusions:**

Our data indicates that Pit-1 regulates MMP-1 and MMP-13, and that inhibition of MMP-13 blocked invasiveness to lung in Pit-1-overexpressed breast cancer cells.

**Electronic supplementary material:**

The online version of this article (doi:10.1186/s13058-014-0505-8) contains supplementary material, which is available to authorized users.

## Introduction

To develop metastasis, breast cancer cells need, among other steps, to break their intercellular adhesion complexes and basement membrane to acquire motility to invade adjacent tissues [1]. Proteolytic enzymes of various classes (metallo, aspartic, cysteine, serine, and threonine) execute the breaking down of matrix elements. However, some components, particularly the interstitial collagens, are very resistant to proteolytic attacks, being degraded only by matrix metalloproteinases (MMPs) [2]. MMPs are synthesized as inactive zymogens, which are then activated predominantly pericellularly by either other MMPs or serine proteases. MMPs’ activity is specifically inhibited by the so-called tissue inhibitors of metalloproteases (TIMPs). Interstitial collagenases are a subfamily of MMPs that cleaves the stromal collagens. This subfamily includes, among others, collagenase 1 (MMP-1), and collagenase 3 (MMP-13). MMP-1 is the most ubiquitously expressed of the interstitial collagenases. It is produced by a wide variety of normal cells, for example, stromal fibroblasts, macrophages, endothelial cells, and epithelial cells, as well as by numerous tumors [3]. MMP-1 is often upregulated in breast cancer, especially in basal-type tumors [4], and seems to be critically involved in metastatic dissemination [5],[6]. Moreover, it has been suggested that MMP-1 is associated with shortened relapse-free survival [7] and poor outcome in breast cancer [4]. Human collagenase-3 (MMP-13) was first identified in breast carcinoma [8]-[10]. Nielsen *et al*. [11] reported that MMP-13 expression by myofibroblasts was often associated with microinvasive events, and they proposed that MMP-13 may play an essential role during the transition from ductal carcinoma *in situ* lesions to invasive ductal carcinoma of the breast.

The POU class 1 homeobox 1 transcription factor (POU1F1, also known as Pit-1) was originally described in the pituitary gland, where it regulates cell differentiation during organogenesis and acts as an activator for pituitary gene transcription [12],[13]. Pit-1 is also expressed in human breast [12]. Compared to normal breast, Pit-1 expression is higher in breast tumors, increases cell proliferation, and regulates the expression of two breast cancer related hormones, growth hormone (GH) and prolactin (PRL) [14]-[16], which are also involved in both MMP regulation and breast cancer metastasis [17],[18]. In addition, Pit-1 overexpression in a mouse xenograft tumor model promotes tumor growth and metastasis in lung. Furthermore, elevated Pit-1 expression in patients with breast cancer is positively correlated with the occurrence of distant metastasis [19].

In the present study, we used human mammary cell lines to analyze the regulation of MMP-1 and MMP-13 by Pit-1. In addition, we used immunodeficient mice to evaluate the role of both metalloproteinases in Pit-1-induced cancer invasiveness. Finally, we evaluated Pit-1, MMP-1, and MMP-13 protein expression in 110 human breast invasive ductal carcinomas.

## Methods

### Cell culture and reagents

The human breast adenocarcinoma cell lines MCF-7 and MDA-MB-231 were obtained from the European Collection of Cell Cultures (ECCC, Salisbury, UK). These cell lines were grown in 100-mm Petri dishes in Dulbecco’s modified Eagle’s medium (DMEM) supplemented with 10% fetal bovine serum (FBS), 100 U/ml penicillin, and 100 μg/ml streptomycin in an air-CO_2_ (95:5) atmosphere at 37°C. Confluent cells were washed twice with phosphate-buffered saline (PBS) and harvested by a brief incubation with trypsin- ethylenediaminetetraacetic acid (EDTA) solution (Sigma-Aldrich, St. Louis, MO, USA). Geneticin (G418), culture medium, and sera were purchased from Invitrogen Life Technologies, Carlsbad, CA, USA. Immobilon-P membranes were from Millipore (Merck Millipore, Billerica, MA, USA). Mytomycin C, MTT, puromycin, and hygromycin B were from Sigma-Aldrich.

### Plasmids and transfections

Transient transfection, Pit-1 knockdown, and stable transfection of Pit-1 into MCF-7 cells were performed as previously described [19],[20]. The Pit-1-overexpressing MCF-7 cells were then transfected with pBABE-puro-Luc vector and 48 hours later treated with 2.5 μg/μl of puromycin to select clones (MCF-7-hPit-1-luc cells). The shpLKO.1-MMP-1 and shpLKO.1-MMP-13 lentiviral vectors containing two different short hairpin RNA (shRNA) sequences for MMP-1 and MMP-13 were obtained from Thermo Fisher Scientific (Waltham, MA, USA) (see Additional file 1 and Figure S1A in Additional file 2).

The pLKO.1-puro non-target shRNA control containing a shRNA (shControl) was obtained from Sigma-Aldrich. MCF-7-hPit-1-luc-shControl, MCF-7-hPit-1-luc-shMMP-1 and MCF-7-hPit-1-luc-shMMP-13 cells were obtained through infection with shControl, shMMP-1, and shMMP-13 virus particles, respectively. Briefly, cell culture medium was replaced by a medium without FBS and containing 5 μg/ml of polybrene.

After 4 hours, culture medium was again replaced (DMEM plus 10% FBS), and lentiviral particles (Mission pLKO.1-puro non-target shRNA, Mission-MMP-1 shRNA, and Mission MMP-13 shRNA transduction particles, Sigma-Aldrich) were added and incubated at 37°C for 24 hours. Pit-1 knockdown was carried out using two different Pit-1 small interfering RNA (siRNA) (Pit-1 siRNA-1 and Pit-1 siRNA-2), as previously described (19). A scrambled siRNA was employed as control. Sequences of siRNAs are detailed in Additional file 1. The proximal promoter regions of the human MMP-1 and MMP-13 genes were synthesized by PCR and the product subcloned into the Xho I and Hind III site of the pGL2-basic plasmid (see Additional file 1). Site-directed mutagenesis was performed with the QuikChange kit from Stratagene (Agilent Technologies, Santa Clara, CA, USA). The mutagenized oligonucleotide primers were as follows (mutagenized bases on the sense strand are identified by lowercase letters): 5′-GATTGCCTAGTCT- AT**gac**TAGCTAATCAAG-3′ and 5′-CCAGGACCCCTG**tcga**CATCTTGAATGG-3′ for MMP-1 and MMP-13, respectively. The newly constructed mutant plasmids were designated pGL2-B-MMP-1-1633/-1MUT and pGL2-B-MMP-13-145/+2MUT.

For luciferase assays, MCF-7 cells were transfected in 6-well plates containing 6 μl of jetPEI Polyplus transfection reagent (PolyPlusTransfection, Illkirch, France), 2 μg of pRc-RSV or pRSV-hPit-1, 1 μg of each reporter plasmid, and 50 ng of pRL-TK-Renilla (as transfection control) for 48 hours. The cells were lysed in buffer (100 μl lysis buffer, Promega Corporation, Madison, WI, USA) and luciferase activity was then measured in a Mithras LB 940 apparatus (Berthold Technologies, Bad Wildbad, Germany).

### RNA isolation and quantitative RT-PCR

Total RNA was isolated from the cell lines using TRIzol (Invitrogen). cDNA was synthesized with Transcriptor First Strand cDNA Synthesis Kit (Roche Diagnostics, Basel, Switzerland), and reactions of quantitative real-time PCR were done using iQ SYBR Green Supermix (Bio-Rad Laboratories, Alcobendas, Spain) on iCycler equipment (7500 PCR Systems, Applied Biosystems, Life Technologies, Carlsbad, CA, USA). The Pit-1, MMP-1, MMP-13 and 18S samples were denatured at 94°C for 10 sec, annealed at 58, 59, 59 and 60°C, respectively, for 10 sec and extended at 72°C for 10 sec, for a total of 33, 35, 35 and 30 cycles, respectively.

The samples were quantified using Sequence Detection Software 1.4 (Applied Biosystems), with 18S as normalization control. The oligonucleotide sequences are described in Additional file 1.

### Cell proliferation (MTT) assay

Cell proliferation experiments were carried out using MTT assay. MCF-7-hPit-1-luc cells, MCF-7-hPit-1-luc-shControl cells, MCF-7-hPit-1-luc-shMMP-1 cells, or MCF-7-hPit-1-luc-shMMP-13 cells (2.5 × 10^4^ cells/ml) were seeded in a volume of 0.5 ml in 24-well tissue culture plates. The absorbance of the samples was recorded 48 hours after transfection at 590 nm in a multiwell plate reader (LB 940 Mithras, Berthold Technologies). Results were plotted as the mean ± SD values of quadruplicates from at least two independent experiments.

### Western blot analysis

Western blotting was carried out as described elsewhere [19]. Briefly, 60 μg of total protein was subjected to SDS-PAGE electrophoresis. Proteins were transferred to a nitrocellulose membrane, blocked, and immunolabeled overnight at 4°C with a primary antibody (detailed in Additional file 1). Then, the membrane was washed three times with PBS-Tween-20, and incubated with the appropriate secondary antibody for 1 hour. The signal was detected with the Pierce enhanced chemiluminescence (ECL) Western blotting substrate (Thermo Fisher Scientific, Rockford, IL, USA), and visualized by placing the blot in contact with standard X-ray film. Relative protein expression was quantified using the ImageJ software (National Institutes of Health, Bethesda, MD, USA) in at least three different blots, and values correspond to mean values of fold-change in relation to beta-actin values.

### ChIP assay

Chromatin immunoprecipitation (ChIP) assays were performed using the Upstate protocol as described previously [20]. Diluted soluble chromatin fractions were immunoprecipitated with 1 μg polyclonal anti-Pit-1 antibody (Santa Cruz Biotechnologies, Heidelberg, Germany), or control human immunoglobulin G (IgG) (Sigma-Aldrich). The histone-DNA crosslinks were reversed by 4-hour incubation at 65°C. PCR was used to analyze the DNA fragments from ChIP assays. Primer sequences are detailed in Additional file 1.

### Wound-healing and cell invasion assays

To perform the wound-healing assay, cells were seeded in 60 mm plates and allowed to reach confluence. Wounding was created using plastic pipette tip, and the cells were serum starved and treated with mitomycin C for 24 and 48 hours. Images were captured by an Olympus DP72 camera (Olympus, Tokyo, Japan), and the distance between the wound edges was measured. Cell invasion assay was performed in BD BioCoat matrigel invasion chambers according to the manufacturer’s instructions (BD Biosciences, San Agustin de Gualix, Spain), as previously described [19]. Uncoated porous filters (8-μm pore size) were used for estimating cell migration, and matrigel-precoated filters were used for examining cell invasion. Values for cell invasion were expressed as the mean number of cells per field over four fields per filter for triplicate experiments.

### Animal studies

All animal studies were approved by the University of Santiago de Compostela Ethics Committee for Animal Experiments. Female mice (age matched, between 6 and 8 weeks) homozygous for the severe combined immune deficiency (SCID) (CB17-*Prkdcscid*, Parc Research Biomedica, Barcelona, Spain) were used for xenografting studies.

Experimental metastasis assays were done as previously described [19]. Briefly, 1 × 10^6^ MCF-7-hPit-1-luc cells (n = 9, controls), MCF-7-hPit-1-luc-shControl cells (n = 8, controls), MCF-7-hPit-1-luc-shMMP-1cells (n = 8), or MCF-7-hPit-1-luc-shMMP-13 cells (n = 8) in 0.15 ml of PBS and matrigel (50:50, BD Biosciences) were injected into the mammary fat pad. At day 24 for control mice or day 33 for MMP-1 and MMP-13 knockdown mice, orthotopic primary mammary tumors were measured (as described below), and removed under anesthesia. Seventeen days later (day 41 after cell injection for controls, and day 50 for MMP-1 and MMP-13 knockdown mice) mice were sacrificed, and lungs removed and examined for metastasis. Xenografts were visualized by luminescence at days 10 (all groups), 24 (Pit-1-overexpressed mice), and 33 (Pit-1-overexpressed and MMP-1 or MMP-13 knockdown) using the In Vivo Imaging System (IVIS, Caliper Life Sciences, Alameda, CA, USA). An intensity map was obtained using the Living Image software (Caliper Life Sciences). The software uses a color-based scale to represent the intensity of each pixel (ranging from blue for low to red for high). Lung micrometastasis was explored in paraffin sections by hematoxylin and eosin (H&E) staining and cytokeratin 7 (CK7) immunostaining. Immunohistochemistry studies were performed in an Autostainer Link 48 (Dako, Glostrup, Denmark). FLEX Ready-to-Use Primary Antibodies to CK7, CK19, and ki-67 (Dako) were used. For detection we used EnVision FLEX/HRP (Dako). The number of metastatic foci was counted in lung after staining with H&E and immunostaining with CK7, and the size of each foci was evaluated by measuring its diameter (in μm) using the Olympus DP-Soft morphometry program in an Olympus DX51 microscope. For automated ki-67 scoring the ACIS III (Automated Cellular Imaging Systems, Dako-Agilent Technologies, Carpinteria, CA, USA) was used. The ACIS III system scans the slide and is capable of differentiating positive and negative nuclei. Six representative areas were selected in each section and the system generated an average score.

### Patients and immunohistochemistry

One hundred and ten patients with invasive breast cancer (without distant metastasis at the time of initial diagnoses) treated at Fundación Hospital de Jove of Gijón (Spain), between 1990 and 2003, were selected based on the availability of clinical history and a minimum 5-year follow-up. The clinicopathological characteristics of patients and their tumors are shown in Table S1 in Additional file 3. Women were treated according to our institutional guidelines. The study adhered to national regulations and was approved by our regional Ethics and Investigation Committee (Comité Ético de Investigación Clínica Regional del Principado de Asturias). Breast carcinoma tissue samples were obtained at the time of surgery. Prior informed consent was obtained from patients. Routinely fixed (overnight in 10% buffered formalin), paraffin-embedded tumor samples stored in our pathology laboratories were used. Histopathologically representative tumor areas without necrosis were defined on H&E-stained sections. Serial 5-μm sections were consecutively cut with a microtome (Leica Microsystems, Barcelona, Spain) and transferred to adhesive-coated slides. Imunohistochemistry was done on these sections using a TechMate TM50 autostainer (Dako) as previously described [19]. A polyclonal anti-Pit-1 (Santa Cruz Biotechnology), monoclonal anti-MMP-1 (NeoMarkers, Fremont, CA, USA) and anti-MMP-13 (Santa Cruz Biotechnology) antibodies were used. To enhance antigen retrieval, tissue sections were treated in a PT-Link™ (Dako) at 97°C for 20 minutes, in citrate buffer pH 6.1 for MMP-1 and in Tris-EDTA buffer pH 9 for MMP-13, and then washed in PBS. Endogenous peroxidase activity was blocked by incubating the slides in peroxidase-blocking solution (Dako) for 5 minutes. The EnVision Detection Kit (Dako) was used as the staining detection system. Sections were counterstained with hematoxylin, dehydrated with ethanol, and permanently coverslipped. For each antibody preparation, the location of immunoreactivity, percentage of reactive area and intensity were determined. All cases were semiquantified for each protein-stained area. An image analysis system with the Olympus BX51 microscope and soft analysis (analySIS™, soft imaging system) were used as follows: tumor sections were stained with antibodies according to the method explained above and counterstained with hematoxylin. There were different optical thresholds for both stains. Each slide was scanned with a 400X power objective and four fields were selected per case to determine protein-reactive areas. The computer program selected and traced a line around antibody-reactive areas (higher optical threshold: red spots), with the remaining, nonstained areas (hematoxylin-stained tissue with lower optical threshold) standing out as a blue background. Area ratios of stained (red) versus nonstained (blue) were determined for all fields. To evaluate immunostaining intensity we used a numeric score from 0 to 3: 0 = no reactivity; 1 = weak reactivity; 2 = moderate reactivity; and 3 = intense reactivity. Using an Excel spreadsheet, the score of one field was obtained by multiplying the intensity score (I) by the percentage of reactivity area (PA) (total score: I × PA). In addition, for each tumor the mean score of the four fields evaluated was calculated. We also evaluated the immunohistochemical staining exclusively in cancerous cells or in stromal cells (mononuclear inflammatory cells, (MICs)- and fibroblast-like cells), and every evaluated field contained at least 10 stromal cells. We considered immunostaining to be positive when at least 10% of cells showed positivity. We distinguished stromal cells from cancer cells because the latter are larger in size, and because fibroblasts are spindle-shaped whereas mononuclear inflammatory cells are rounded. Moreover, while cancer cells are arranged forming either acinar or trabecular patterns, stromal cells are spread.

### Statistical analysis

Values are expressed as mean ± standard deviation (SD). Means were compared using two-tailed Student’s *t* test or one-way ANOVA, with the Tukey-Kramer multiple comparison test for *post hoc* comparisons. After analyzing the human tumor distribution of score values by the Kolmogorov-Smirnov test, nonparametric methods were used to analyze the data. Immunostaining score values for each protein were expressed as a median (range). Correlation between score values was calculated by using the Spearman correlation test. Comparison of immunostaining values between groups was done with the Mann-Whitney or Kruskal-Wallis tests. Statistical results were corrected applying Bonferroni’s correction. *P* values of less than 0.05 were considered statistically significant. The PASW Statistics 18 program was used for all calculations (SPSS Inc, Chicago, IL, USA).

## Results

### Pit-1 regulates MMP-1 and MMP-13 mRNA and protein levels in MCF-7 and MDA-MB-231 cell lines

**Figure 1:**
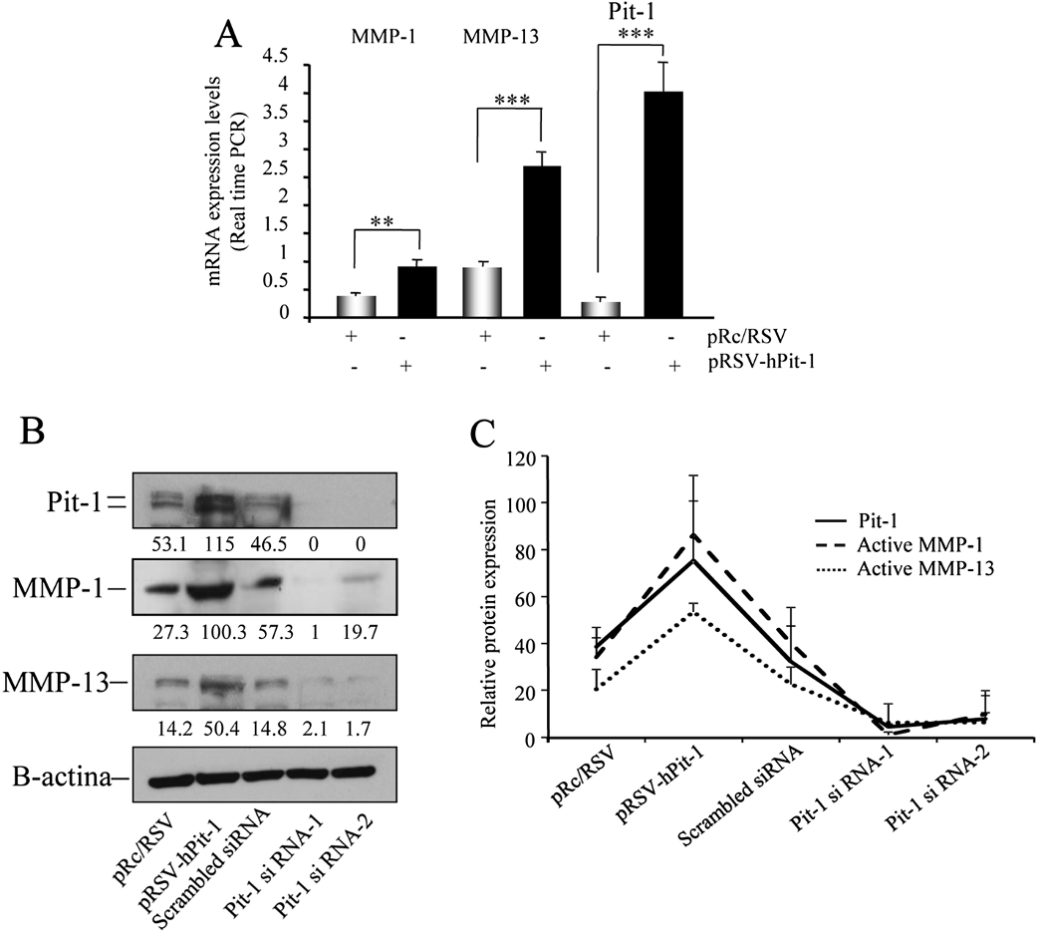
**Pit-1 regulates MMP-1 and MMP-13 expression. (A)** MCF-7 cells were transfected with the pRSV-hPit-1 overexpression vector and 48 hours later a real-time PCR was carried out to evaluate MMP-1, MMP-13, and Pit-1 mRNA expression. **(B)** Western blot of MMP-1, MMP-13, Pit-1 and β-actin in MDA-MB-231 cells 48 hours after Pit-1 overexpression or Pit-1 knockdown. **(C)** Densitometric quantitation of three different blots. Values expressed as mean ± SD represent relative Pit-1, MMP-1, and MMP-13 protein expression in relation to β-actin values. MMP-1, matrix metalloproteinase-1; MMP-13, matrix metalloproteinase-13; Pit-1, POU class 1 homeobox 1.

To evaluate the effect of Pit-1 on MMP-1 and MMP-13 mRNA expression, we carried out a real-time PCR. Pit-1 overexpression in MCF-7 cells using the pRSV-hPit-1 vector induced a significant increase in both MMP-1 (*P* <0.01) and MMP-13 (*P* <0.001) mRNA signal in relation to controls (Figure 1A). Our data indicated that Pit-1 overexpression increased MMP-1 and MMP-13 mRNA between two and three times, respectively. Given that MMP protein levels in MCF-7 cell extracts are low, MMP-1 and MMP-13 protein expression was evaluated in MDA-MB-231 cells by Western blot. Figure 1B shows increased and decreased MMP-1 and MMP-13 protein expression after Pit-1 overexpression and Pit-1 knockdown, respectively. Three different blots were quantified by densitometry, and the results are shown in Figure 1C.

### Regulation of MMP-1 and MMP-13 by the Pit-1 transcription factor at transcriptional level

**Figure 2:**
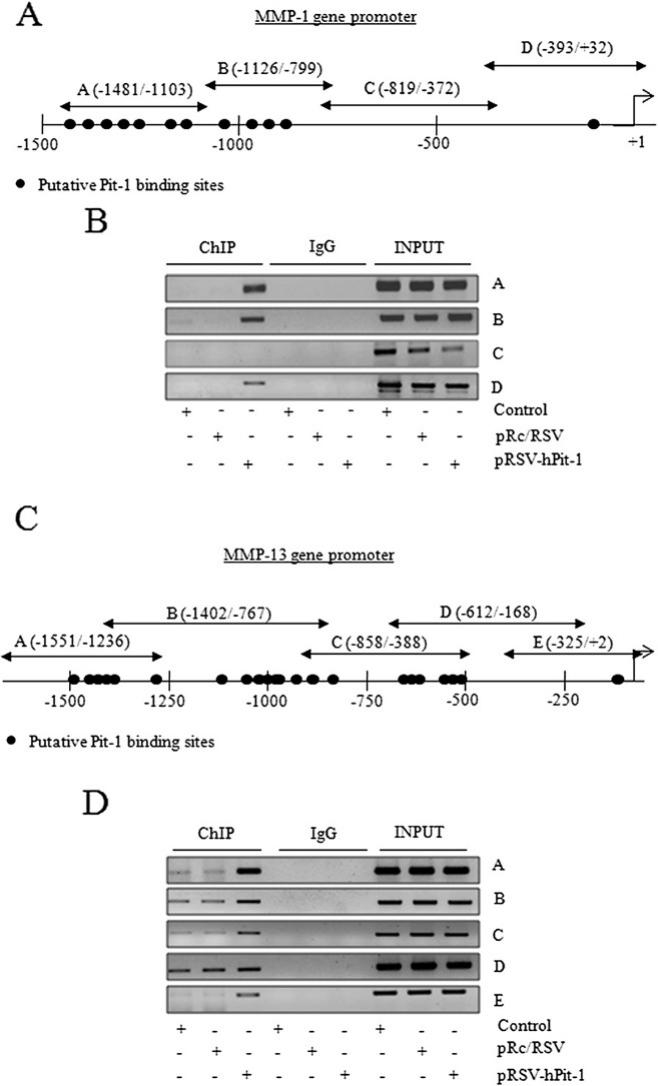
**Pit-1 binds to the MMP-1 and MMP-13 promoter. (A)** Diagram of the human MMP-1 gene promoter showing the putative Pit-1 binding sites, and the location of the primers used in the ChIP assay. **(B)** Soluble chromatin prepared from control, pRc/RSV, or pRSV-hPit-1-transfected MCF-7 cells were immunoprecipitated with an anti-Pit-1 antibody or control IgG. The immunoprecipitated DNA was amplified by PCR using primers (A, B, C, and D) that amplified regions of the MMP-1 promoter with or without the putative Pit-1 binding sites. **(C)** Diagram of the human MMP-13 gene promoter showing the putative Pit-1 binding sites, and the location of the primers used in the ChIP assay. **(D)** Soluble chromatin and immunoprecipitation were performed as described in B. The immunoprecipitated DNA was amplified by PCR using primers (A, B, C, D, and E) that amplified regions of the MMP-13 promoter. ChIP, chromatin immunoprecipitation; MMP-1, matrix metalloproteinase-1; MMP-13, matrix metalloproteinase-13; Pit-1, POU class 1 homeobox 1.

**Figure 3:**
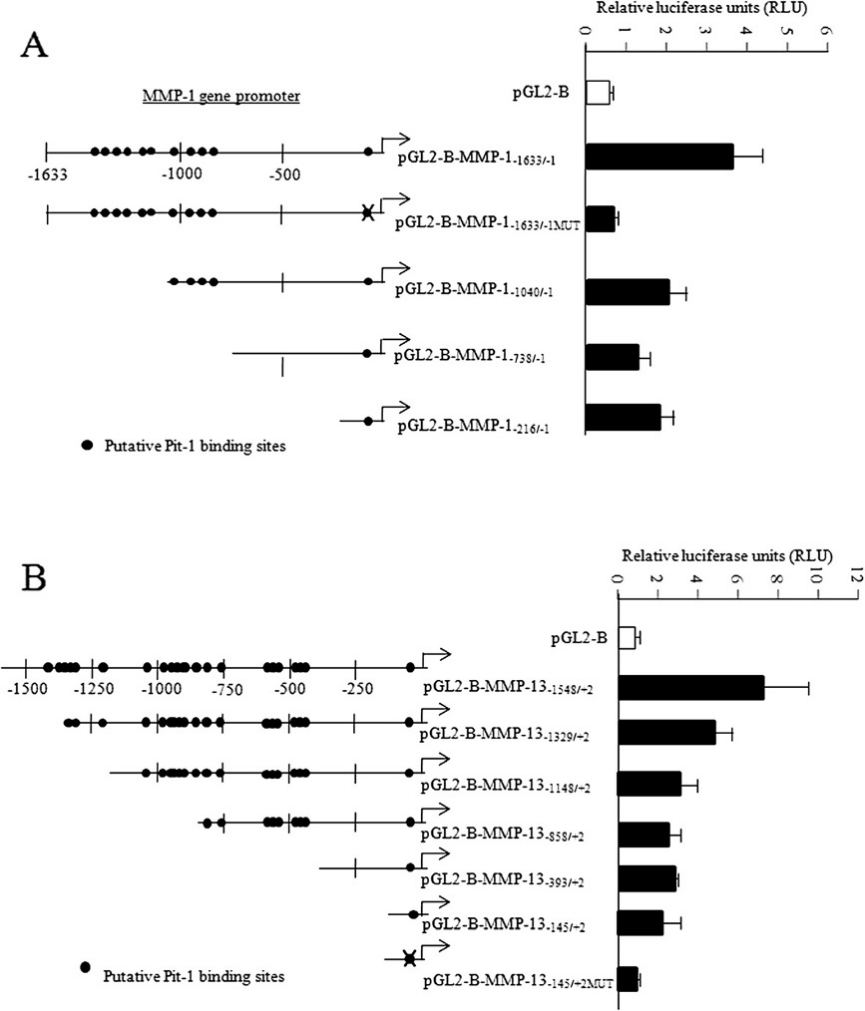
**Deletion analysis identified a Pit-1-responsive region in the human MMP-1 and MMP-13 promoter. (A)** The MMP-1 promoter fragments that fused to the pGL2-Basic vector (pGL2B) were transfected into MCF-7 cells and then transfected with the pRc/RSV or the pRSV-hPit-1 vector. Normalized relative luciferase units (RLU) were calculated as the ratio of luciferase activity in the pRSV-hPit-1-transfected cells to that in the corresponding control (pRc/RSV-transfected) cells. **(B)** The MMP-13 promoter fragments that fused to the pGL2Basic vector (pGL2B) were transfected as indicated in A. Data are expressed as indicated in A. MMP-1, matrix metalloproteinase-1; MMP-13, matrix metalloproteinase-13; Pit-1, POU class 1 homeobox 1.

Using the Transcription Element Search Software program (TESS, University of Pennsylvania, Philadelphia, PA, USA [21]), several putative Pit-1 binding sites were found in the MMP-1 and MMP-13 promoter (Figure 2A-C). To determine whether Pit-1 binds to the MMP-1 and MMP-13 genes, a ChIP assay was carried out using gene-specific primers (see Additional file 1). Specific binding of Pit-1 to the A, B, and D sites of the MMP-1 promoter was observed in pRSV-hPit-1-overexpressed MCF-7 cells, indicating that Pit-1 binds to the MMP-1 promoter gene *in vivo* (Figure 2B). A ChIP assay was also carried out to evaluate Pit-1 binding to the MMP-13 promoter (Figure 2D). Our data indicated specific Pit-1 binding to regions A, B, C, D, and E in the MMP-13 promoter, suggesting MMP-13 regulation by Pit-1, such as occurs with the MMP-1 gene. Transfection reporter assays (Figure 3A-B) indicate that Pit-1 transcriptionally regulates the MMP-1 and the MMP-13 gene. Specific deletions of the MMP-1 promoter demonstrate that Pit-1 binds to a region comprised between −1633/-1 bp from the start transcription site and this region is necessary for Pit-1-induced MMP-1 transcription. The functionality of the proximal Pit-1 response element was tested by mutation in the context of the pGL3B-MMP-1-1633/-1 construct (see Additional file 1). Specifically, three different mutations were introduced into the MMP-1-1633/-1 sequence at a position −104/-101 from the transcription start site (pGL3B-MMP-1-1633/-1MUT). This construct was cotransfected into MCF-7 cells with the pRc/RSV or with the pRSV-hPit-1 vector. As shown in Figure 3A, the response to Pit-1 was completely wiped out in cells transfected with the mutant construct. Similarly, we also evaluated transcriptional regulation of the MMP-13 promoter by Pit-1 using several constructs containing specific deletions of the pGL3B-MMP-13-1548/+2 construct. As observed in Figure 3B, the response to Pit-1 overexpression in MCF-7 cells gradually decreased, depending on the size of the MMP-13 promoter. Mutation of a Pit-1 binding site in the position −35/-31 bp in the pGL2-B-MMP-13-145/+2MUT construct (see Additional file 1) reduced transcriptional activation as compared with the wild-type construct (pGL2-B-MMP-13-145/+2). In summary, our data suggest that Pit-1 regulates MMP-1 and MMP-13 expression by binding to their gene promoter region.

### Knockdown of MMP-1 and MMP-13 reduces motility and invasion in Pit-1-overexpressing cells

**Figure 4:**
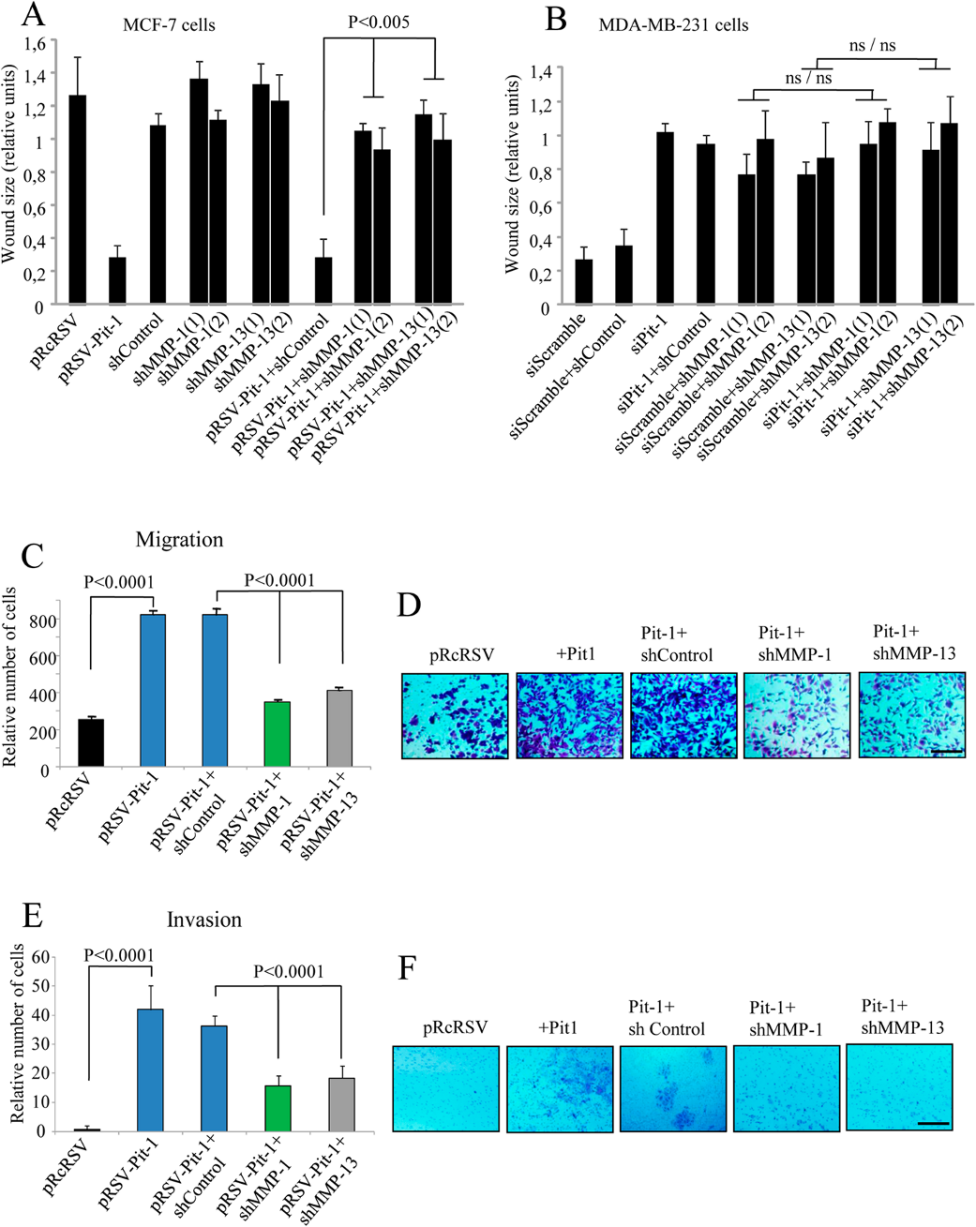
**MMP-1 and MMP-13 knockdown reduces invasive features in MCF-7 cells with Pit-1 overexpression, and in MDA-MB-231 cells. (A-B)** Wound-healing assay in (A) MCF-7 cells with Pit-1 overexpression (pRSV-hPit-1), and knockdown of MMP-1 (shMMP-1(1) and shMMP-1(2)) and MMP-13 (shMMP-13(1) and shMMP-13(2)); (B) MDA-MB-231 cells with knockdown of Pit-1 (siPit-1), MMP-1 (shMMP-1(1) and shMMP-1(2)), and MMP-13 (shMMP-13(1) and shMMP-13(2)). Distance between the wound edges was measured at 48 hours in three different assays, and data are represented as mean ± SD; ns = not significant. **(C-D)** Cell motility through uncoated filters (migration) at 24 hours in control MCF-7 cells (pRcRSV), Pit-1-overexpressing MCF-7 cells (pRSV-hPit-1), and Pit-1-overexpressing and knockdown of MMP-1 or MMP-13 MCF-7 cells (pRSV-hPit-1 + shMMP-1 or −13). **(E-F)** Cell motility through matrigel-coated filters at 48 hours in control cells, cells transfected with the pRSV-hPit-1 vector, and cells transfected with pRSV-hPit-1 and knockdown of MMP-1 (Pit-1 + shMMP-1) or MMP-13 (Pit-1 + shMMP-13). Numbers represent mean ± SD. Scale bar: 100 μm. MMP-1, matrix metalloproteinase-1; MMP-13, matrix metalloproteinase-13; Pit-1, POU class 1 homeobox 1.

To evaluate the effect of MMP-1 and MMP-13 knockdown on motility and invasion in MCF-7 cells with Pit-1 overexpression and in MDA-MB-231 cells with Pit-1 knockdown, we carried out wound-healing, migration, and invasion assays. As previously demonstrated, Pit-1 induces a significant increase in MCF-7 cell migration [19]. This was also observed in the present study, as demonstrated by the absence of wound after Pit-1 overexpression (Figure 4A and Figure S2 in Additional file 4). Knockdown of both MMP-1 and MMP-13 (two independent hairpins) significantly (*P* <0.005) reduced cell motility in Pit-1-overexpressing MCF-7 cells (Figure 4A, and Figure S2 in Additional file 4). In MDA-MB-231 cells, which have higher basal Pit-1 expression levels than MCF-7 cells, Pit-1 knockdown significantly reduced cell motility (Figure 4B and Figure S3 in Additional file 5). However, in Pit-1 knocked-down cells, neither MMP-1(1) and (2) nor MMP-13(1) and (2) knockdown significantly modified migration as compared to cells with MMP-1(1) and (2) and MMP-13(1) and (2) knockdown alone (Figure 4B, and Figure S3 in Additional file 5). We next explored whether MMP-1 and MMP-13 knockdown affected migration and invasion in Pit-1-overexpressing MCF-7 cells in the matrigel invasion assay. Figures 4C-F shows that knockdown of either MMP-1 or MMP-13 led to a significant (*P* <0.0001) decrease in both migration and invasion capacity of Pit-1-overexpressed MCF-7 cells, as compared with only Pit-1-overexpressed MCF-7 cells. We also studied the effect of MMP-1 and MMP-13 knockdown on three-dimensional growth. MDA-MB-231 cells were cultured in matrigel, in which the cells form spherical structures. However, reduced levels of MMP-1 or MMP-13 expression did not modify three-dimensional growth of MDA-MB-231 cells, as compared with control cells (Figure S1 B-C in Additional file 2).

### MMP-13 knockdown in a xenograft mice model with Pit-1 overexpression blocked breast cancer invasiveness

**Figure 5:**
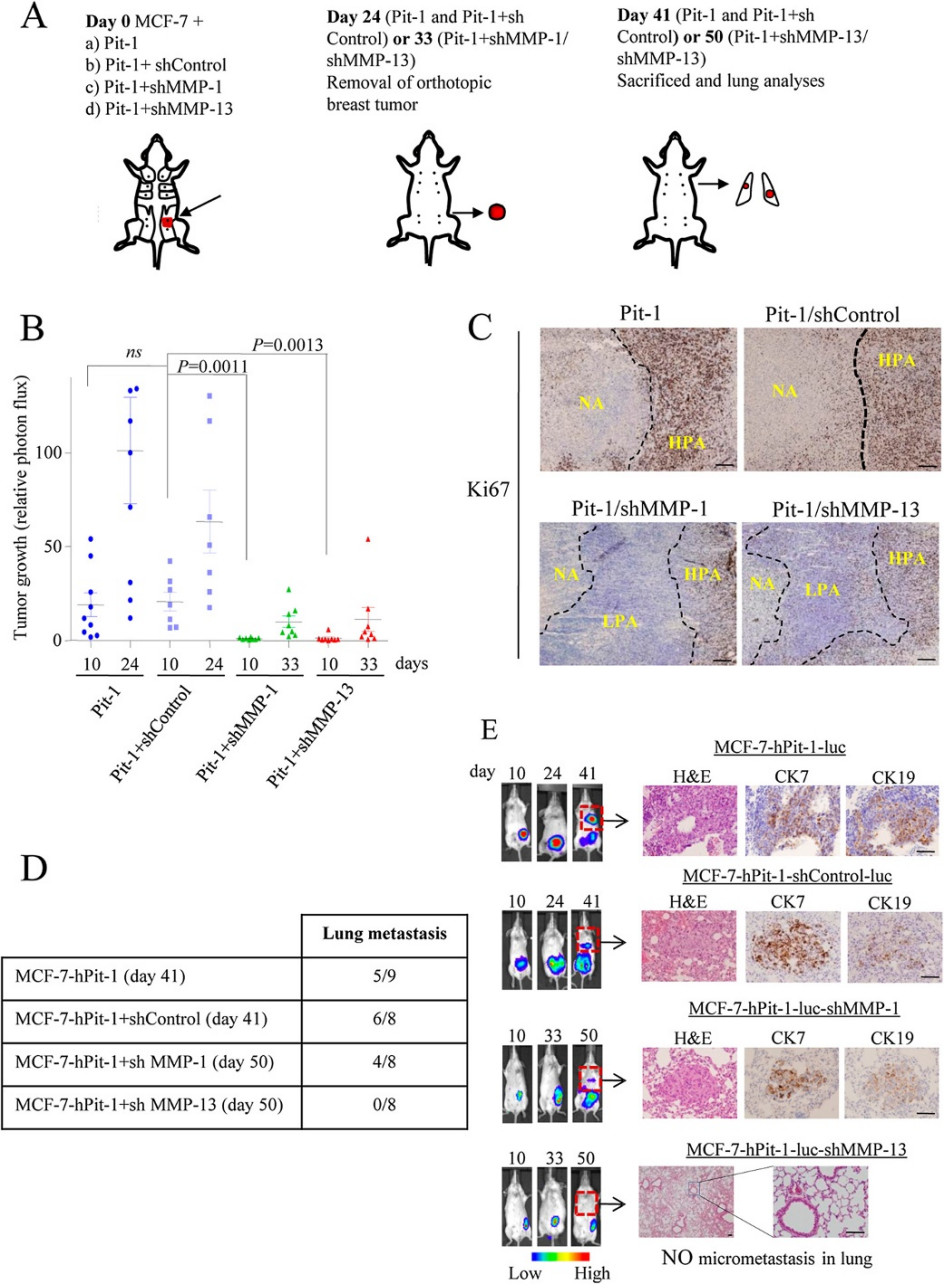
**Orthotopic injection of MCF-7 cells with Pit-1 overexpression and MMP-13 knockdown in SCID mice blocks metastasis to lung. (A)** Schematic representation of experimental induction of metastasis. At day 0, SCID mice were injected into the mammary fat pad either with MCF-7 cells: (a) with Pit-1 overexpression (controls, n = 9), (b) with Pit-1 overexpression and shControl (controls, n = 8), (c) with Pit-1 overexpression and MMP-1 knockdown (n = 8), and (d) with Pit-1 overexpression and MMP-13 knockdown (n = 8). At day 24 (in mice with Pit-1 overexpression and shControl) or day 33 (in MMP-1 and MMP-13 knockdown mice), animals were anesthetized and breast tumors resected. Mice lived until day 41 (Pit-1 overexpression) or day 50 (Pit-1 plus MMP-1 or MMP-13 knockdown), and then were sacrificed and lungs removed for analysis. **(B)** Scatter plots of tumor growth in SCID mice at days 10 (all groups), 24 (mice with Pit-1 overexpression, Pit-1, and Pit-1 + shControl), and 33 (mice with Pit-1 overexpression and MMP-1 or MMP-13 knockdown), as described in A. Horizontal bars represent mean ± SEM. **(C)** ki67 immunostaining of tumors from mice injected with MCF-7 and Pit-1 overexpression (Pit-1), Pit-1 + shControl, Pit-1 + shMMP-1, or Pit-1 + shMMP-13. NA: necrotic area; HPA: high proliferative area; LPA: low proliferative area. Scale bar: 50 μm. **(D)** Five out of the nine mice with Pit-1 overexpression (day 41), six out of the eight with Pit-1 overexpression and shControl (day 41), and four out of the eight with Pit-1 overexpression and MMP-1 knockdown (day 50) developed lung metastasis, while none of the eight mice with MMP-13 knockdown (day 50) showed micrometastasis in lung. **(E)** Representative example of mice. Color indicates tumor cell luminescence. H&E staining and CK-7 and CK-19 immunopositivity in lung. Scale bar: 100 μm. CK, cytokeratin; H&E, hematoxylin and eosin; MMP-1, matrix metalloproteinase-1; MMP-13, matrix metalloproteinase-13; Pit-1, POU class 1 homeobox 1; SCID, severe combined immunodeficiency.

It has been shown that MCF-7 cells present very low levels of metastasis [22],[23]. However, overexpression of Pit-1 in this cell line significantly increases metastasis in lung [19]. Therefore, we tested the potential to metastasize *in vivo* of MCF-7 cells stably transfected with Pit-1 alone or with Pit-1 and either MMP-1 or MMP-13 knockdown. SCID female mice were injected in the mammary fat pad with either MCF-7-hPit-1-luc cells (n = 9), MCF-7-hPit-1-luc-shControl cells (n = 8), MCF-7-hPit-1-luc-shMMP-1(1) cells (n = 8), or MCF-7-hPit-1-luc-shMMP-13(1) cells (n = 8). Twenty-four days after MCF-7-hPit-1-luc and MCF-7-hPit-1-luc-shControl cell injection, and 33 days after MCF-7-hPit-1-luc-shMMP-1 and -shMMP-13 injection, primary mammary tumors were excised under anesthesia in both groups of animals (Figure 5A). Our data indicated that knockdown of MMP-1 and MMP-13 reduced tumor growth in Pit-1-overexpressing mice. In fact, tumor growth at day 10 was significantly lower in mice injected with MCF-7-hPit-1-luc-shMMP-1 (*P* = 0.0011) and -shMMP-13 (*P* = 0.0013) cells as compared to controls (Figure 5B). At day 24, considerable tumor growth was observed in controls, while almost no tumor growth was observed in MMP-1 and MMP-13 knockdown mice (data not shown). Even though tumors in knockdown mice were allowed to continue growing until day 33, only very slight growth was found (Figure 5B). To evaluate why the growth of tumors in mice with Pit-1 overexpression was faster than in mice with Pit-1 overexpression plus MMP-1 or MMP-13 knockdown, we evaluated the ki-67 proliferation marker expression in tumors by immunohistochemistry. As shown in Figure 5C, tumors from mice injected with MCF-7-hPit-1-luc and MCF-7-hPit-1-luc-shControl cells had a larger proliferative area (high ki-67 expression, 83.6 ± 13.7% and 93.0 ± 3.7%, respectively) and a larger necrotic area than those injected with MCF-7-hPit-1-luc-shMMP-1 and -shMMP-13, which showed low ki-67 expression (47.2 ± 19.6% and 36.2 ± 26.3%, respectively). This suggests that the small tumor size in MMP-1 and MMP-13 knockdown mice could be due to low cell proliferation. An example of whole tumor size stained with ki-67 is shown in Figure S4A in Additional file 6. In addition, we carried out an *in vitro* MTT assay using MCF-7 cells to evaluate cell proliferation. Our data show a significant (*P* <0.001) decrease in cell proliferation at 48 hours in cells with Pit-1 overexpression and MMP-1 or MMP-13 knockdown as compared to cells with Pit-1 overexpression alone (Figure S4B in Additional file 6).

After excision of tumors, mice lived until day 41 (Pit-1 and Pit + shControl) and 50 (Pit-1 + shMMP-1 or shMMP-13) after injection of MCF-7 cells. Mice were then sacrificed and lungs were removed, fixed, paraffin-embedded, sectioned, and stained with H&E and specific antibodies for immunohistochemistry analysis. Five out of the nine mice injected with Pit-1-overexpressing cells (controls), six out of the eight mice injected with Pit-1-overexpressing cells + shControl, and four out of the eight mice injected with Pit-1-overexpressing + shMMP-1 transfected cells developed micrometastases in lung, while none of the eight mice injected with the MCF-7 cells transfected with the pTRE2-hPit-1-Luc-shMMP-13 showed micrometastases in lungs (Figure 5D). Micrometastases in lungs showed immunopositivity for CK-7, and CK-19 (Figure 5E). These antibodies react only with human and not with mouse cytokeratins. Neither size (Pit-1: 166.2 ± 174.1 μm, Pit-1 + shControl: 183.7 ± 79.2 μm, and Pit-1 + shMMP-1: 185.5 ± 132.1 μm) nor number of metastases (Table S2 in Additional file 7) were significant among groups of mice.

### Expression of both MMP-1 and MMP-13 correlates with Pit-1 expression in human breast tumors

**Figure 6:**
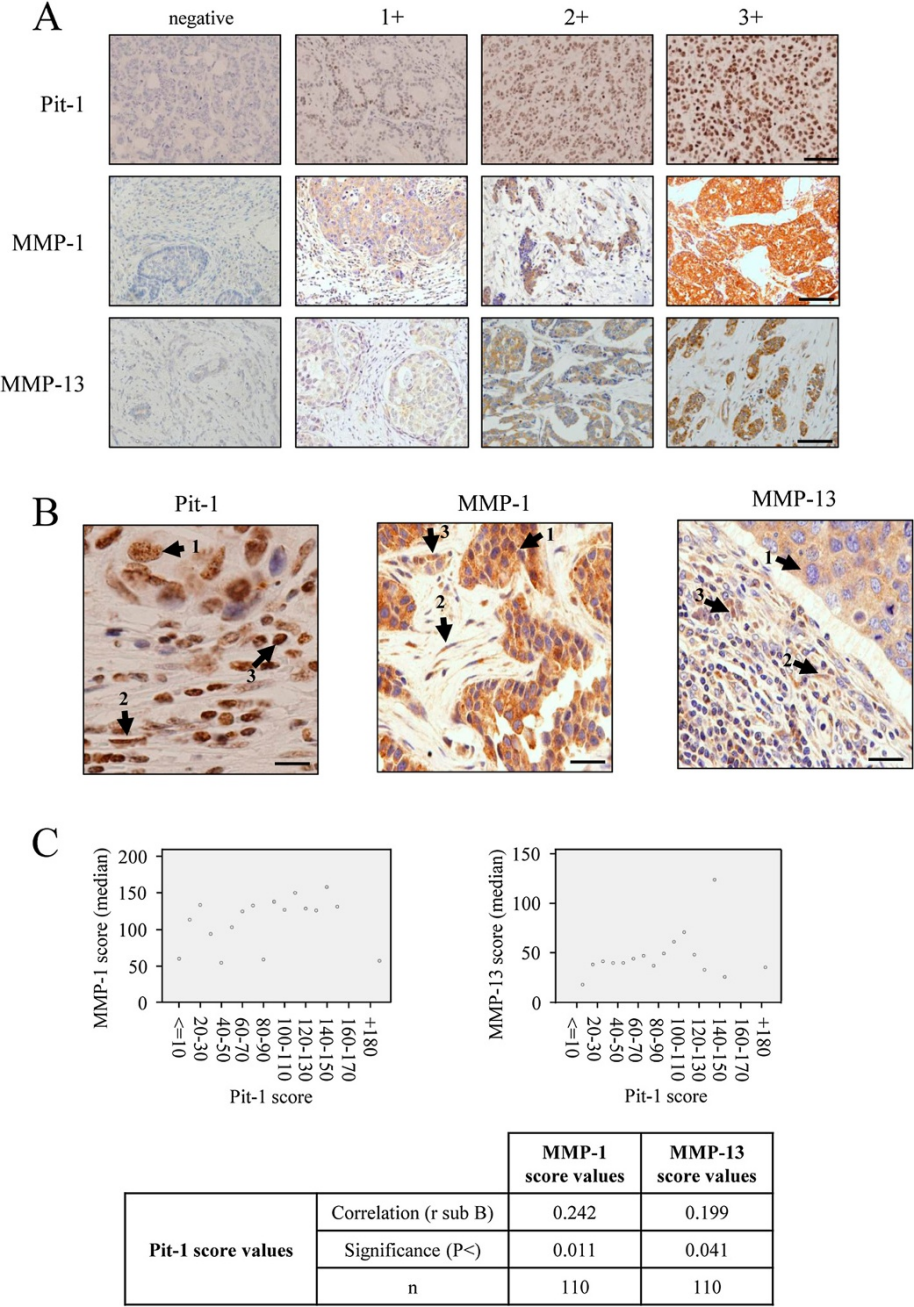
**Pit-1 expression in human ductal invasive carcinomas of the breast positively correlates with MMP-1 and MMP-13 expression. (A)** Representative set of negative and positive (1+, 2+, and 3+) Pit-1, MMP-1, and MMP-13 immunodetection in human breast invasive ductal carcinomas. Scale bar: 100 μm. **(B)** Representative figures of positive Pit-1, MMP-1, and MMP-13 expression in tumor cells (1), fibroblasts (2), and mononuclear inflammatory cells (MIC, 3). **(C)** Plots of median score values of MMP-1 and MMP-13 as a function of Pit-1 score groups, and table with the values of Spearman correlation test. n = 110 human breast tumors. MMP-1, matrix metalloproteinase-1; MMP-13, matrix metalloproteinase-13; Pit-1, POU class 1 homeobox 1.

To further evaluate the clinical value of MMP-1, MMP-13 and Pit-1 expression in human breast tissue, these proteins were analyzed by immunohistochemistry in 110 invasive ductal carcinomas of the breast. Representative examples of Pit-1, MMP-1 and MMP-13 immunostaining in tumors are shown in Figure 6A. Pit-1 protein expression was detected mainly in the nuclei of tumor or control epithelial cells, whereas MMP-1 and MMP-13 expression had a cytoplasmic location in all positive cases. MMP-1 and MMP-13 expression was also evaluated in stromal cells (that is fibroblasts, and mononuclear inflammatory cells, MICs) and correlated with global Pit-1 expression. Although Pit-1 expression was not quantified by cell group, it was indeed observed in all three cell groups. An example of Pit-1, MMP-1, and MMP-13 expression in tumor and stromal cells is shown in Figure 6B. The percentage of positive cells for each protein was always higher than 50% in positive case for each cell type. A total of 89 tumors (80.9%) stained positively for Pit-1, with clear differences in intensity and percentage of stained cells. MMP-1 expression was detected in 105 tumors (95.4%) and MMP-13 in 84 tumors (76.4%). The median score value was 35.4 (range 0 to 194.5) for Pit-1, 119 (0 to 206.2) for MMP-1, and 42 (0 to 135.8) for MMP-13. Distribution of Pit-1, MMP-1 and MMP-13 score values are shown in Figure S5 in Additional file 8. In relation to global expression (score values) of Pit-1, MMP-1 and MMP-13, our result showed a direct correlation between Pit-1 score values and MMP-1 (r sub B = 0.242, *P* = 0.011) and MMP-13 (r sub B = 0.199, *P* = 0.041) (Figure 6C). In addition, we observed that MMP-1 and MMP-13 global expression was higher in Pit-1-positive tumors compared with Pit-1-negative tumors (median (range): MMP-1: 123.7 (0 to 206.2) vs. 60.4 (0 to 151.8), *P* = 0.014; MMP-13: 43.9 (0 to 135.8) vs. 0 (0 to 67.9), *P* = 0.034). A significant positive correlation between Pit-1 and both MMP-1 and MMP-13 was also found in human breast tumor datasets [24]-[26].

Our results also showed significant associations between proteins in terms of cellular type. Score values of Pit-1 were significantly higher in tumors with MMP-1-positive fibroblasts and MMP-13-positive fibroblasts than in tumors with MMP-1-negative fibroblasts and MMP-13-negative fibroblasts (*P* = 0.017 and *P* = 0.029, respectively) (Table 1). Likewise, score values of Pit-1 were significantly higher in tumors with MMP-1-positive cancerous cells and MMP-13-positive cancerous cells than in tumors with MMP-1- or MMP-13-negative cancerous cells (*P* <0.001) (Table 1).

**Table 1:** **Relationships between MMP-1 and MMP-13 expression by each cell type and Pit-1 global expression in 110 breast carcinomas**

Factor	N	Pit-1 median (range)	*P* value
**MMP-1**			
Tumor cell			**0.004**
*negative*	5	5.6 (0 – 13.1)	
*positive*	105	38.3 (0 – 194.5)	
Fibroblast			**0.017**
*negative*	15	21.5 (0 – 89.6)	
*positive*	95	27.4 (0 – 194.5)	
MICs			0.068
*negative*	30	31.8 (0 – 124.5)	
*positive*	80	36.7 (0 – 194.5)	
**MMP-13**			
Tumor cell			**0.0001**
*negative*	26	12.2 (0 – 135.6)	
*positive*	84	45.1 (0 – 194.5)	
Fibroblast			**0.029**
*negative*	60	32.5 (0 – 194.5)	
*positive*	50	43.9 (0 – 143.2)	
MICs			0.120
*negative*	82	33.8 (0 – 194.5)	
*positive*	28	45.0 (0 – 132.3)	

MMP-1, matrix metalloproteinase-1; MMP-13, matrix metalloproteinase-13; Pit-1, POU class 1 homeobox 1; MICs: mononuclear inflammatory cells.

In addition, we evaluated the potential association between score values from Pit-1, MMP-1 and MMP-13 and relapse-free survival in all patients included in the present study. The determination of optimal cutoff values for Pit-1 and MMP-1 in breast tumors was done for predicting recurrence. *P* values obtained for each cutoff value are plotted against the value itself (Figure S6A-B in Additional file 9). This statistical analysis showed no significant association between score values of MMP-13 and recurrence (data not shown). However, our data showed that high score values for Pit-1 and MMP-1 were significantly associated with recurrence. This analysis led us to define a score value of 22 for Pit-1 (χ 2 = 4.71, *P* = 0.03) and of 130 for MMP-1 (χ 2 = 6.52, *P* = 0.011) as optimal cutoff points. These values identified 73 (66.4%) and 40 (36.4%) patients, respectively, with a high probability of recurrence (Figure S6C-D in Additional file 9). We also determined the relapse-free survival curves for patients with breast carcinomas based on the combination of these optimal cutoff points. Both high Pit-1 and MMP-1 expression identified a patient subgroup (n = 31, 28.2%) with the highest probability of recurrence (*P* = 0.004) (Figure S6E in Additional file 9). The relationship between Pit-1, MMP-13 expression and prognosis was determined in MICs. Breast tumors containing MICs that were positive for Pit-1 and MMP13 (n = 24, 21.8%) had a high probability of recurrence (*P* = 0.010) (Figure S6F in Additional file 9).

## Discussion

In this study we found that Pit-1 regulated MMP-1 and MMP-13 in breast cancer cells at transcriptional level. Our data indicated that knockdown of MMP-13 blocked mammary cancer invasiveness to lung in xenografts with Pit-1 overexpression. In addition MMP-1 and MMP-13 positive expression in fibroblasts and tumor cells is positively correlated with high Pit-1 score values in human breast tumors.

The role of Pit-1 in breast carcinogenesis has recently been demonstrated [19]. Pit-1 overexpression in the mammary fat pads of SCID mice is related with metastasis in lung, and elevated levels of Pit-1 in node-positive breast cancer patients are positively correlated with distant metastasis. Interestingly, moderately elevated Pit-1 expression levels were found in 36% of patients with invasive ductal carcinoma of the breast [19]. However, the mechanism of Pit-1 metastasis induction is unknown. Given that MMPs are key proteins involved in the metastatic process, in the present study we evaluated the role of Pit-1 on the expression and biological activity of two collagenases, MMP-1 and MMP-13, whose role in several processes of the metastatic disease is well known [3]. In the MCF-7 and MDA-MB-231 human breast cancer cell lines, we showed that Pit-1 regulates MMP-1 and MMP-13. This regulation is carried out at transcriptional level, as demonstrated by ChIP and luciferase reporter assays. Knockdown of either MMP-1 or MMP-13 significantly reduced motility and invasion capacity of MCF-7 cells with Pit-1 overexpression. These are relevant findings considering that MMP-1 and MMP-13 have exceptionally wide substrate specificity when compared with other MMPs, and because these molecules are implicated in the degradation of the connective stromal tissue and invasion of the basement membranes, which are key actions in the metastatic process. MMP-1 cleaves several components of the extracellular matrix, including collagen type I (the principal component of the connective tissue), II, III, VII, VIII, and IX, aggrecan, as well as serine protease inhibitors, and α2 macroglobulin [3],[27]. MMP-13 efficiently degrades the native helix of fibrillar collagens with preferential activity on type II collagen [28],[29]. However, MMP-13 is also able to degrade several other extracellular matrix proteins *in vitro*, including collagens type IV, X, and XIV; fibronectin; tenascin; and fibrillin [30],[31]. In addition, it has been shown that MMP-13 plays a central role in the MMP activation cascade, both activating and being activated by other MMPs (MMP-14, 2 or 3) [32].

In order to explore whether knockdown of each of these MMPs could also modify the metastatic potential of Pit-1-overexpressing MCF-7 cells *in vivo*, we evaluated the effect of MMP-1 and MMP-13 knockdown in an SCID mice tumor xenograft model. Our data indicated that knockdown of either MMP-1 or MMP-13 reduced cell proliferation, as demonstrated by the low ki-67 expression in tumors compared to controls. Probably as a consequence of this, tumor growth was also decreased in xenografts. This was previously demonstrated with MMP-13 in the human squamous cell carcinoma xenografts [33], and with MMP-1 in the human breast carcinoma cell line MDA-MB-231 in the mammary fat pad xenograft model [34], and recently in the MMP-1a knockout mouse, which has significantly decreased lung tumor growth and angiogenesis [35]. On the contrary, overexpression of MMP-1 (in conjunction with other genes) in human breast carcinoma cells increased xenograft growth rates [5], and facilitated the assembly of new tumor blood vessels, the release of tumor cells into the circulation, and the breaching of lung capillaries by circulating tumor cells to seed pulmonary metastasis [36]. In human patients, it has recently been demonstrated that MMP-1 expression by MICs from sentinel lymph nodes (SLNs) was significantly associated with metastatic spread to non-SLNs, suggesting that the degradation capacity of MMP-1 in the extracellular matrix may be responsible for promoting tumor spread via the lymph nodes [37].

Our data indicate that MMP-13 knockdown completely blocked cancer cell invasiveness to lung, suggesting that MMP-13 is a necessary mediator of Pit-1 induction of breast metastasis to lung. This experimental finding could be in line with previous clinical data indicating that MMP-1 and MMP-13 seem to be related with different metastatic profiles in breast cancer. Whereas MMP-13 expression (but not MMP-1 expression) was significantly and independently associated with the occurrence of distant metastasis in breast cancer [7],[38], MMP-1 expression was strongly associated with the metastatic progression across the axillary lymphatic system [37]. These data seem to support the hypothesis that hematogenous metastasis and regional lymph node metastasis are different processes of tumor spread [39],[40], which may require different substrate-specific degradation.

In the present study we also found positive correlations between global expression (score values) of Pit-1 and either MMP-1 or MMP-13 expression in human primary breast carcinomas. Nevertheless, the great complexity of interactions between cancerous cells and stromal cells in the context of these malignancies should be taken into account. Thus, for example, it has been described that MMP-13 is produced by fibroblast-like cells located in the stromal compartment of the breast cancer tissue [41], whereas other studies have indicated that MMP-13 is synthesized predominantly by epithelial tumor cells [8],[42]. In our study, both MMP-1 and MMP-13 expression was observed in all three cell types studied (tumor cells, fibroblasts, and MICs), but we only found a significant correlation between MMP-1 and MMP-13 positive expression and high Pit-1 levels in tumor cells and fibroblasts. It is well known that tumor stroma play a fundamental role in tumor growth, invasion and dissemination, and that fibroblasts are the prevailing component of tumor stroma [7],[43]-[45]. Thus, it is tempting to speculate that high Pit-1 levels in fibroblasts from breast tumors could induce increased MMP-1 and MMP-13 expression, which in turn may increase collagen degradation and facilitate the dissemination of tumor cells to lung. However, only MMP-13 knockdown blocks dissemination of Pit-1-overexpressing tumor cells to lung, suggesting that MMP-13 mediates in this process.

In summary, our data indicates that Pit-1 increases and activates MMP-1 and MMP-13 expression acting at transcriptional level by binding to their promoters. In mice, knockdown of MMP-13 blocks Pit-1-induced breast cancer cell invasiveness induced by Pit-1. Finally, in human breast tumors there is a significant correlation between Pit-1 and MMP-1 and MMP-13 expression in both tumor and fibroblast cells, suggesting a relationship between Pit-1 and MMPs expression in Pit-1-induced metastasis to lung.

## Conclusions

Breast cancer is a heterogeneous illness that encompasses several distinct disease entities, often referred to intrinsic subtypes of breast cancer. It has previously been demonstrated that patients with breast cancer and overexpression of the Pit-1 transcription factor are associated with higher occurrence of distant metastasis, but the mechanisms remain unknown. The present study demonstrates that Pit-1 increases MMP-1 and MMP-13 expression at transcriptional level. Given that these MMPs have been related to breast cancer metastasis, we explored the effect of Pit-1 overexpression and MMP-1 or MMP-13 knockdown in an SCID mouse xenograft tumor model. Both Pit-1 overexpression and Pit-1 overexpression together with MMP-1 knockdown induced metastasis in lung. On the other hand, Pit-1 overexpression and MMP-13 knockdown completely blocked breast cancer invasiveness to lung. We further showed that Pit-1 positively correlated with MMP-1 and MMP-13 expression in 110 human breast tumors, and positive Pit-1 expression also correlated with positive expression of MMP-1 and MMP-13 in tumor cells and fibroblasts. Taken together, our data point to MMP-13 as a target in breast tumors with Pit-1 overexpression.

## Additional files

## Electronic supplementary material

Additional file 1: Supplementary methods (plasmids and transfections, siRNAs, ChIP assay, real-time PCR, antibodies). (PDF 154 KB)

Additional file 2: Figure S1.: Effect of MMP-1 or MMP-13 knockdown on three-dimensional (3D) growth in MDA- MB-231 cell culture. **(A)** MMP-1 and MMP-13 protein expression after MMP-1 and MMP-13 knockdown. MDA-MB-231 cells were transfected with MMP-1 shRNA (1), MMP-1 shRNA (2), MMP-13 shRNA (1) and MMP-13 shRNA (2) and 48 hours later protein extracts were evaluated by Western blot. **(B-C)** Three-dimensional (3D) growth of MDA-MB-231 cells after MMP-1 and MMP-13 knockdown. MDA-MB-231 cells transfected with missense control shRNA, or shMMP-1 (B) or shMMP-13 (C) were cultured in solidified matrigel for 10 days and phase contrast photographs of cells as monolayers or in three-dimensional (3D) cultures were taken with an Olympus DP72 camera. The quantitation of sphere diameter was performed manually by tracing a straight line across the diameter of the sphere and scoring its value as arbitrary length units. Scale bar: 75 mm. (PDF 180 KB)

Additional file 3: Table S1.: Basal characteristics of 110 patients with invasive ductal carcinoma of the breast. (PDF 74 KB)

Additional file 4: Figure S2.: Wound healing was carried out in MCF-7 cells with Pit-1 overexpression (pRSV-hPit-1), and knockdown of MMP-1 (shMMP-1(1) and shMMP-1(2)) and MMP-13 (shMMP-13(1) and shMMP-13(2)). Wounding was done using plastic pipette tip. At 24 and 48 hours, the distance between the wound edges was measured. Images were captured with an Olympus DP72 camera. Scale bar: 150 μm. (PDF 684 KB)

Additional file 5: Figure S3.: Wound healing was carried out in MDA-MB-231 cells with Pit-1 knockdown (siPit-1), and knockdown of MMP-1 (shMMP-1(1) and shMMP-1(2)) and MMP-13 (shMMP-13(1) and shMMP-13(2)). Wounding was done using plastic pipette tip. At 24 and 48 hours, the distance between the wound edges was measured. Images were captured with an Olympus DP72 camera. Scale bar: 150 μm. (PDF 757 KB)

Additional file 6: Figure S4.: MPP-1 and MMP-13 knockdown reduces tumor size. **(A)** Photos of slides showing tumors size at day 24 (in mice with Pit-1 overexpression, Pit-1, and Pit-1 + shControl) or day 33 (in mice injected with MCF-7-hPit-1-luc-shMMP-1 cells, or MCF-7-hPit-1- luc-shMMP-13 cells, Pit-1 + shMMP-1 and Pit-1shMMP-13, respectively), and immunostained with ki-67. Scale bar: 1 cm. **(B)** Cell proliferation (MTT) assay in MCF-7 control cells, MCF-7-hPit-1-luc cells, MCF-7-hPit-1-luc-shControl cells, MCF-7-hPit-1-luc-shMMP-1 cells, and MCF-7-hPit-1-luc-shMMP-13 cells. The absorbance of the samples was measured 48 h after transfection. Results were plotted as the mean ± SD values of quadruplicates from at least two independent experiments. (PDF 43 KB)

Additional file 7: Table S2.: MMP-13 but not MMP-1 knockdown blocks metastasis in lung. **(A)** Number of metastasis in lung at day 41 (in MCF-7-hPit-1-luc- and MCF-7-hPit-1-luc-shControl-injected mice) or day 50 (in MCF-7-hPit-1-luc-shMMP-1-injected mice). Number was quantified after CK7 staining. Size represents mean + SD of total metastasis measurements. **(B)** Size (diameter in μm of each metastasis) and number of metastasis (specified for each mice) was evaluated using the Olympus DP-Soft morphometry program in an OlympusDX51 microscope. (PDF 186 KB)

Additional file 8: Figure S5.: Distribution score values obtained by immunohistochemical staining of Pit-1, MMP-1, and MMP-13 in 110 invasive ductal carcinomas of the breast. (PDF 232 KB)

Additional file 9: Figure S6.: Determination of cutoff values and their relationship with biochemical recurrence. Maximum likelihood determination of **(A)** Pit-1 and **(B)** MMP-1 cutoff values for predicting biochemical recurrence in 110 patients with breast cancer. The values obtained for each cutoff value are plotted against the value itself. Statistical significance is indicated by the horizontal line at 0.05. Analysis led to the definition of 22 for Pit-1 (χ 2 = 4.71, *P* = 0.03) and 130 for MMP-1 (χ 2 = 6.52, *P* = 0.011) as the optimal cutoff points. Probability of relapse-free survival as a function of the optimal cutoff point for **(C)** Pit-1 score values (*P* = 0.03), **(D)** MMP-1 (*P* = 0.011), and **(E)** the combination of both cutoff points (*P* = 0.004). **(F)** Probability of relapse-free survival as a function of Pit-1 and MMP-13 expression by inflammatory mononuclear cells. (PDF 210 KB)

Below are the links to the authors’ original submitted files for images.

Authors’ original file for figure 1

Authors’ original file for figure 2

Authors’ original file for figure 3

Authors’ original file for figure 4

Authors’ original file for figure 5

Authors’ original file for figure 6

## Abbreviations

cDNA: complementary DNA
ChIP: chromatin immunoprecipitation
CK7: cytokeratin 7
DMEM: Dulbecco’s modified Eagle’s medium
ECL: enhanced chemiluminescence
EDTA: ethylenediaminetetraacetic acid
FBS: fetal bovine serum
GH: growth hormone
H&E: hematoxylin and eosin
IgG: immunoglobulin G
MIC: mononuclear inflammatory cell
MMP-1: matrix metalloproteinase-1
MMP-13: matrix metalloproteinase-13
mRNA: messenger RNA
PBS: phosphate-buffered saline
PCR: polymerase chain reaction
Pit-1: POU class 1 homeobox 1
POU1F1: POU class 1 homeobox 1
PRL: prolactin
SCID: severe combined immunodeficiency
SDS-PAGE: sodium dodecyl sulfate polyacrylamide gel electrophoresis
shRNA: small hairpin RNA
siRNA: small interfering RNA
SLN: sentinel lymph node
TIMPs: tissue inhibitors of metalloproteases
