# Erratum to: Cancer progression by breast tumors with Pit-1-overexpression is blocked by inhibition of metalloproteinase (MMP)-13

**DOI:** 10.1186/s13058-017-0834-5

**Published:** 2017-03-28

**Authors:** Juan Sendon-Lago, Samuel Seoane, Noemi Eiro, Maria A. Bermudez, Manuel Macia, Tomas Garcia-Caballero, Francisco J. Vizoso, Roman Perez-Fernandez

**Affiliations:** 1 Department of Physiology- Center for Research in Molecular Medicine and Chronic Diseases (CIMUS), School of Medicine, University of Santiago de Compostela, 15782 Santiago de Compostela, Spain; 2 Unidad de Investigación, Fundacion Hospital de Jove, 33290 Gijón, Spain; 3 Departments of Obstetrics and Gynecology, School of Medicine, University of Santiago de Compostela, 15782 Santiago de Compostela, Spain; 4 Departments of Morphological Sciences, School of Medicine, University of Santiago de Compostela, 15782 Santiago de Compostela, Spain

## Erratum

After the publication of this work [1] an error was noticed in Fig. 4d and 4f. In the migration and invasion assays the same image was used accidentally for the Pit-1 + shMMP-1 and Pit-1 + shMMP-13. The corrected figure is shown below. The error does not affect the findings or conclusion of the article. We apologize for this error.

**Fig. 4:**
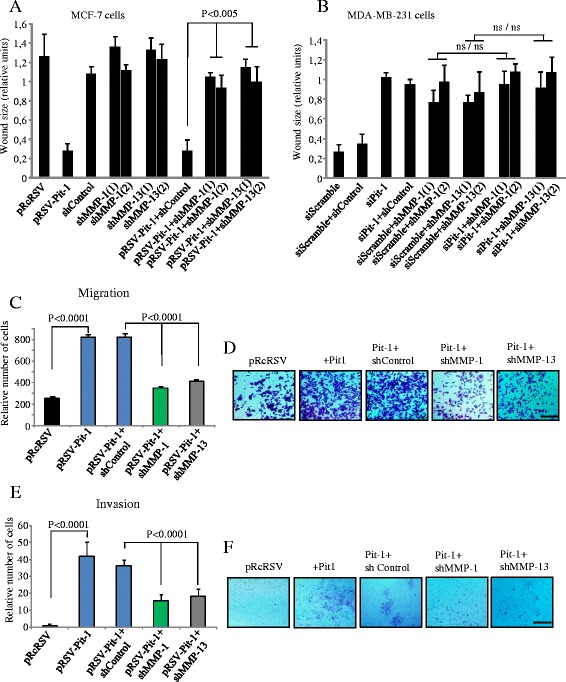
MMP-1 and MMP-13 knockdown reduces invasive features in MCF-7 cells with Pit-1 overexpression, and in MDA-MB-231 cells. **a-b** Wound-healing assay in (**a**) MCF-7 cells with Pit-1 overexpression (pRSV-hPit-1), and knockdown of MMP-1 (shMMP-1(1) and shMMP-1(2)) and MMP-13 (shMMP-13(1) and shMMP-13(2)); (B) MDA-MB-231 cells with knockdown of Pit-1 (siPit-1), MMP-1 (shMMP-1(1) and shMMP-1(2)), and MMP-13 (shMMP-13(1) and shMMP-13(2)). Distance between the wound edges was measured at 48 hours in three different assays, and data are represented as mean ± SD; ns = not significant. **c-d** Cell motility through uncoated filters (migration) at 24 hours in control MCF-7 cells (pRcRSV), Pit-1-overexpressing MCF-7 cells (pRSV-hPit-1), and Pit-1-overexpressing and knockdown of MMP-1 or MMP-13 MCF-7 cells (pRSV-hPit-1 + shMMP-1 or −13). **e-f** Cell motility through matrigel-coated filters at 48 hours in control cells, cells transfected with the pRSV-hPit-1 vector, and cells transfected with pRSV-hPit-1 and knockdown of MMP-1 (Pit-1 + shMMP-1) or MMP-13 (Pit-1 + shMMP-13). Numbers represent mean ± SD. Scale bar: 100 μm. MMP-1, matrix metalloproteinase-1; MMP-13, matrix metalloproteinase-13; Pit-1, POU class 1 homeobox 1
