# Supplementary material for: Cancer progression by breast tumors with Pit-1-overexpression is blocked by inhibition of metalloproteinase (MMP)-13
Source: Breast Cancer Res. 2014 Dec 20;16:505. doi: 10.1186/s13058-014-0505-8 (PMC4305241; doi:10.1186/s13058-014-0505-8)
Supplement: Supplementary file 1 — Additional file 1: Supplementary methods (plasmids and transfections, siRNAs, ChIP assay, real-time PCR, antibodies). (PDF 154 KB) [file 13058_2014_505_MOESM1_ESM.pdf]

Additional File 1

Plasmids and transfections

The shMMP-1 and shMMP-13 sequences were the following:

	**Oligonucleotide sequences**
shpLKO.1-MMP-1(1)	5’-CCGG-TGAAGATGAAAGGTGGACCAACTCGAGTT GGTCCACCTTTCATCTTCA-TTTTT-3'
shpLKO.1-MMP-1(2)	5'-CCGG-AGCACATGACTTTCCTGGAATCTCGAGATTC CAGGAAAGTCATGTGCT-TTTTT-3'
shpLKO.1-MMP-13(1)	5'-CCGG-CCAACCGTATTGATGCTGCATCTCGAGATGCA GCATCAATACGGTTGG-TTTTTG-3'
shpLKO.1-MMP-13(2)	5'-CCGG-GCTCCGAGAAATGCAGTCTTTCTCGAGAAAGA CTGCATTTCTCGGAGC-TTTTTG-3'

The MISSION pLKO.1-puro Non-Target shRNA Control Transduction Particles (Sigma-Aldrich) containing a shRNA insert that does not target any known genes from any species was used as control.

The proximal promoter regions of the human MMP-1 gene (pGL2-B-MMP-1$_{-1633/-1}$, pGL2-B-MMP-1$_{-1040/-1}$, pGL2-B-MMP-1$_{-738/-1}$ and pGL2-B-MMP-1$_{-216/-1}$) were synthesized by PCR using the following oligonucleotides:

	**Oligonucleotide sequences**
pGL2-B-MMP-1$_{-1633/-1}$ (Fw)	5′-CCGCTCGAGGAAATTGTAGTTAAATAATT-3
pGL2-B-MMP-1$_{-1040/-1}$ (Fw)	5´-CCGCTCGAGAGCCTTTTCATCATCCGGTA-3´
pGL2-B-MMP-1$_{-738/-1}$ (Fw)	5´-CCGCTCGAGCATGACTCCATGCTTGGCTA-3´
pGL2-B-MMP-1$_{-216/-1}$ (Fw)	5´-CCGCTCGAGGAGTGTGTCTCCTTCGCACA-3´
Reverse primer (-20/-1)	5′-CCCAAGCTTATCCCAGCTAGGAAGCTCCC-3′

The proximal promoter regions of the human MMP-13 gene (pGL2-B-MMP-13$_{-1548/+2}$, pGL2-B-MMP-13$_{-1329/+2,}$ pGL2-B-MMP-13$_{-1148/+2}$, pGL2-B-MMP-13$_{-858/+2,}$ pGL2-B-MMP-13$_{-393/+2}$, pGL2-B-MMP-1$_{-145/-1}$) were synthesized by PCR using the following oligonucleotides:

	**Oligonucleotide sequences**
pGL2-B-MMP-13$_{-1548/+2}$ (Fw)	5′- CCGCTCGAGCATCAAAATGGGCATCTCCT-3′
pGL2-B-MMP-13$_{-1329/+2}$ (Fw)	5´-CCGCTCGAGGGAACCAAGAATTAGAATAT-3´
pGL2-B-MMP-13$_{-1148/+2}$ (Fw)	5´-CCGCTCGAGTCAGGGGTGTAATTCACAATTC-3´
pGL2-B-MMP-13$_{-858/+2}$ (Fw)	5´-CCGCTCGAGTGCTAAAATTTAGATTACATTGA-3´
pGL2-B-MMP-13$_{-393/+2}$ (Fw)	5´-CCGCTCGAGTGACATCTAAGGTGTTATCGTCAA-3´

pGL2-B-MMP-1$_{-145/+2}$ (Fw)	5´-CCGCTCGAGTGCAAACTGGAGGTCTTCCT-3´
Reverse primer (-18/+2)	5′-CCCAAGCTTATCAACAGTCCCCAGGCATC-3′

Pit-1 siRNA
For silencing Pit-1, MCF-7 cells were transfected with two different Pit-1 siRNA (Pit-1 siRNA-1, Pit-1 siRNA-2). A scrambled siRNA was used as control. Primer sequences were the following:

	**Oligonucleotide sequences**
Pit-1 siRNA-1	site 1, 5'-AACCCCTTGTCTTTACAAGTTCCTGTCTC-3' (antisense)<br>site-2, 5'-AATTAAGTTAGGATACACCCACCTGTCTC-3' (antisense)<br>site-3, 5'-AATTGAATCTCGAGAAAGAAGCCTGTCTC-3' (antisense)
Pit-1 siRNA-2	site-1, 5'-AATGTTGCTGTAGACATCACACCTGTCTC-3´ (antisense)<br>site-2, 5´-AAGCTTTCAGTTTGCATGCATCCTGTCTC- 3´ (antisense)<br>site-3, 5´ -AACTTTCTCCAGATTCAGTTCCCTGTCTC- 3´ (antisense)
Scrambled siRNA	5'-AAGCTTCATAAGGCGCATAGC-3'

CHIP assay
MMP-1 and MMP-13 primer sequences were as follows:

	**Oligonucleotide sequences**
MMP-1	(A) Sense: 5´-ACAGTGGGAAAGGTGGGAGAC-3´<br>Antisense 5´-GTCTATCTTCGCTCCTTGGTGCT-3´<br>(B) Sense 5´-AGCACCAAGGAGCGAAGATAGAC-3´<br>Antisense 5´- GAGGCTGCATTGAGCTGTGA- 3´<br>(C) Sense 5´-TCACAGCTCAATGCAGCCTC-3´<br>Antisense 5´- CAGGGCAGAGGGTGGAATTAC-3´<br>(D) Sense 5´-GTAATTCCACCCTCTGCCCTG-3´<br>Antisense 5´- GTGATGGCTTCCCAGCCTCT-3´
MMP-13	(A) Sense: 5´-CATCATCAAAATGGGCATCT-3´<br>Antisense 5´-ATGATGTCAGCAATGCCATC-3´<br>(B) Sense 5´-GCACAGGTGTTTGGTAAATTAGTG-3´<br>Antisense 5´-TGGAACTCTTCATCTTGAGCA-3´<br>(C) Sense 5´-TGCTAAAAGTTTAGATTACATTGA-3´<br>Antisense 5´-GCATCTTGGCTTTTTCATGA-3´<br>(D) Sense 5´-TCTCGGCAACCATATTAAAGCT-3´<br>Antisense 5´-ATGTACCAACCGCATCATCA-3´<br>(E) Sense 5´-GGAGCCTCTCAGTCATGGAG-3´<br>Antisense 5´-GATGCCTGGGGACTGTTGAT-3´.

The length of MMP-1 PCR products were 378 pb (A), 327 pb (B), 447 pb (C), and 425 pb (D). The length of MMP-13 PCR products were 315 pb (A), 635 pb (B), 470 pb (C), 444 pb (D), and 327 pb (D).

Real-Time PCR
The oligonucleotide sequences were as follows:

	Oligonucleotide sequences
Pit-1	Sense: 5´-GTGTCTACCAGTCTCCAACC-3´<br>Antisense: 5´-ACTTTTCCGCCTGAGTTCCT-3
MMP-1	Sense: 5´-ATGCTGAAACCCTGAAGGTG -3´<br>Antisense: 5´-CTGCTTGACCC- TCAGAGACC -3´
MMP-13	Sense: 5´-TAAGGAGCATGGCGACTTCT-3´<br>Antisense: 5´-GGTCCTTGGAG-TGGTCAAGA-3´
18S	Sense: 5'-GTAACCCGTTGAACCCCATT-3'<br>Antisense: 5'-CCATCCAATCGCTA GTAGCG-3'

Antibodies

Primary antibodies	Antibody dilution	Origin	Secundary antibody
MMP-1 (polyclonal)	1:1000	Millipore	Anti-rabbit
MMP-13 (polyclonal)	1:1000	Santa Cruz Biotechnology	Anti-rabbit
Pit-1 (polyclonal)	1:500	Santa Cruz Biotechnology	Anti-rabbit
β-Actin (monoclonal)	1:5000	Millipore	Anti-mouse
Ki-67 (MIB-1 clon)	1:200	Dako	Anti-mouse
CK7 (clon OV-TL12/30)	1:100	Dako	Anti-mouse
CK19 (clon RKC108)	1:100	Dako	Anti-mouse
