# Supplementary figures and images for: Cancer progression by breast tumors with Pit-1-overexpression is blocked by inhibition of metalloproteinase (MMP)-13
Source: Breast Cancer Res. 2014 Dec 20;16:505. doi: 10.1186/s13058-014-0505-8 (PMC4305241; doi:10.1186/s13058-014-0505-8)

A

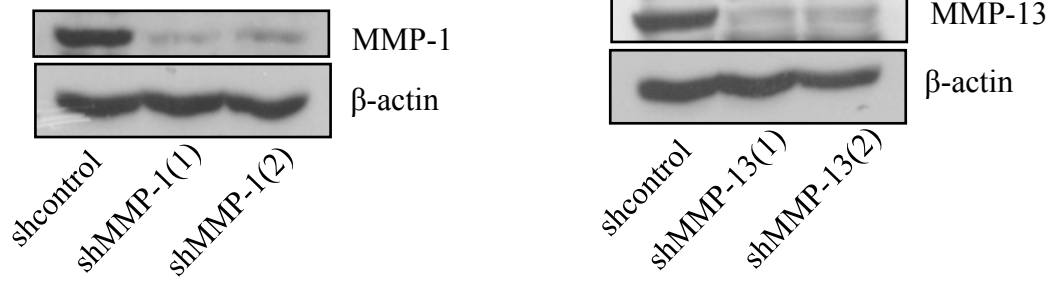

B

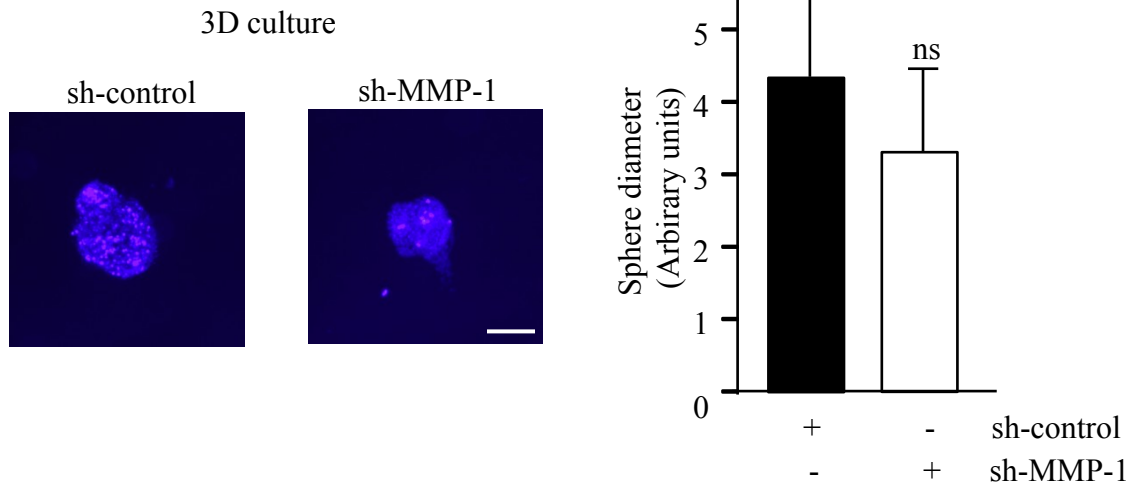

C

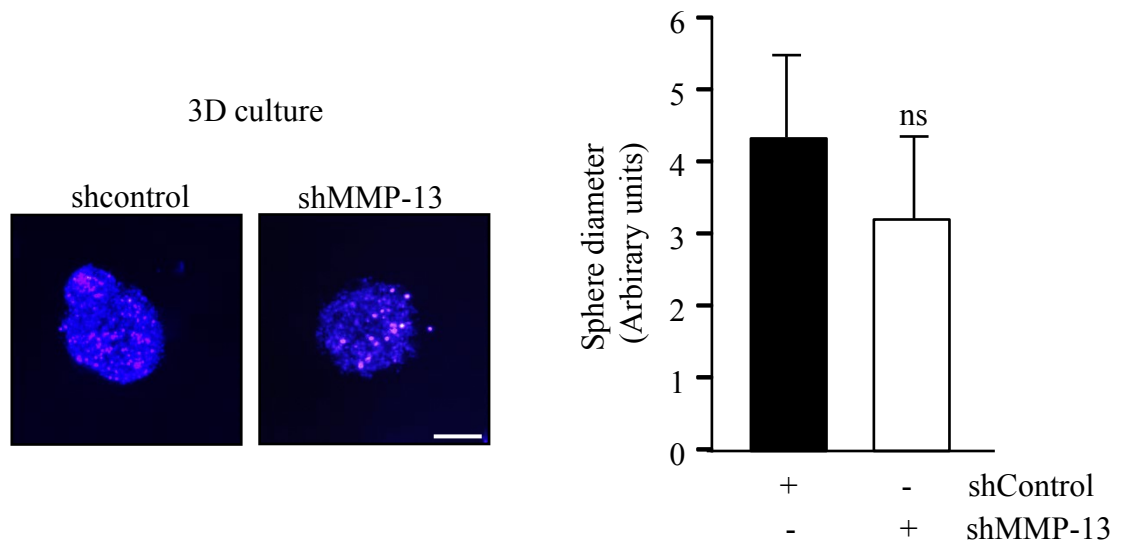

Supplement: Supplementary file 2 — Additional file 2: Figure S1.: Effect of MMP-1 or MMP-13 knockdown on three-dimensional (3D) growth in MDA- MB-231 cell culture. (A) MMP-1 and MMP-13 protein expression after MMP-1 and MMP-13 knockdown. MDA-MB-231 cells were transfected with MMP-1 shRNA (1), MMP-1 shRNA (2), MMP-13 shRNA (1) and MMP-13 shRNA (2) and 48 hours later protein extracts were evaluated by Western blot. (B-C) Three-dimensional (3D) growth of MDA-MB-231 cells after MMP-1 and MMP-13 knockdown. MDA-MB-231 cells transfected with missense control shRNA, or shMMP-1 (B) or shMMP-13 (C) were cultured in solidified matrigel for 10 days and phase contrast photographs of cells as monolayers or in three-dimensional (3D) cultures were taken with an Olympus DP72 camera. The quantitation of sphere diameter was performed manually by tracing a straight line across the diameter of the sphere and scoring its value as arbitrary length units. Scale bar: 75 mm. (PDF 180 KB) [file 13058_2014_505_MOESM2_ESM.pdf]

MCF-7 cells

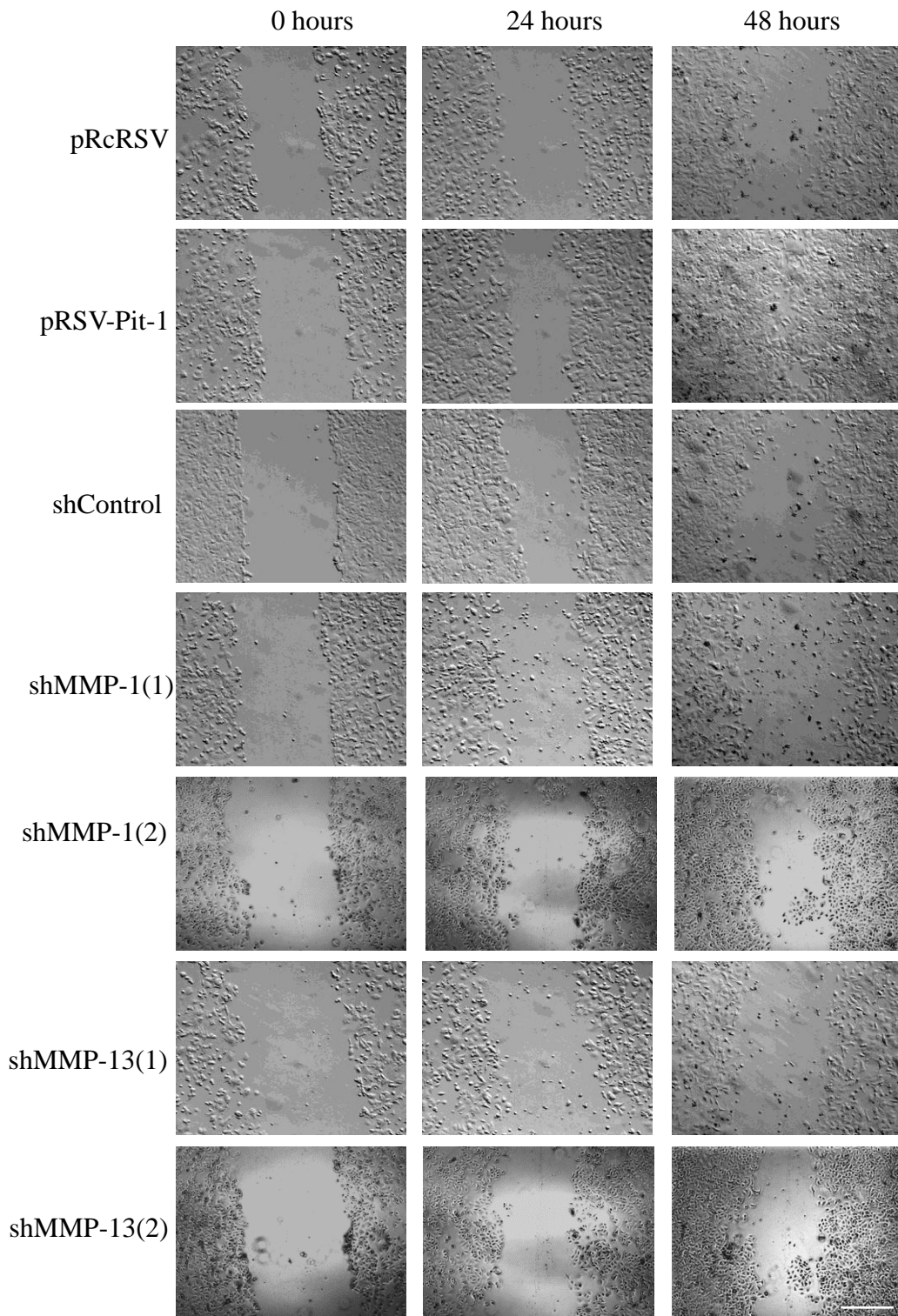

## MCF-7 cells

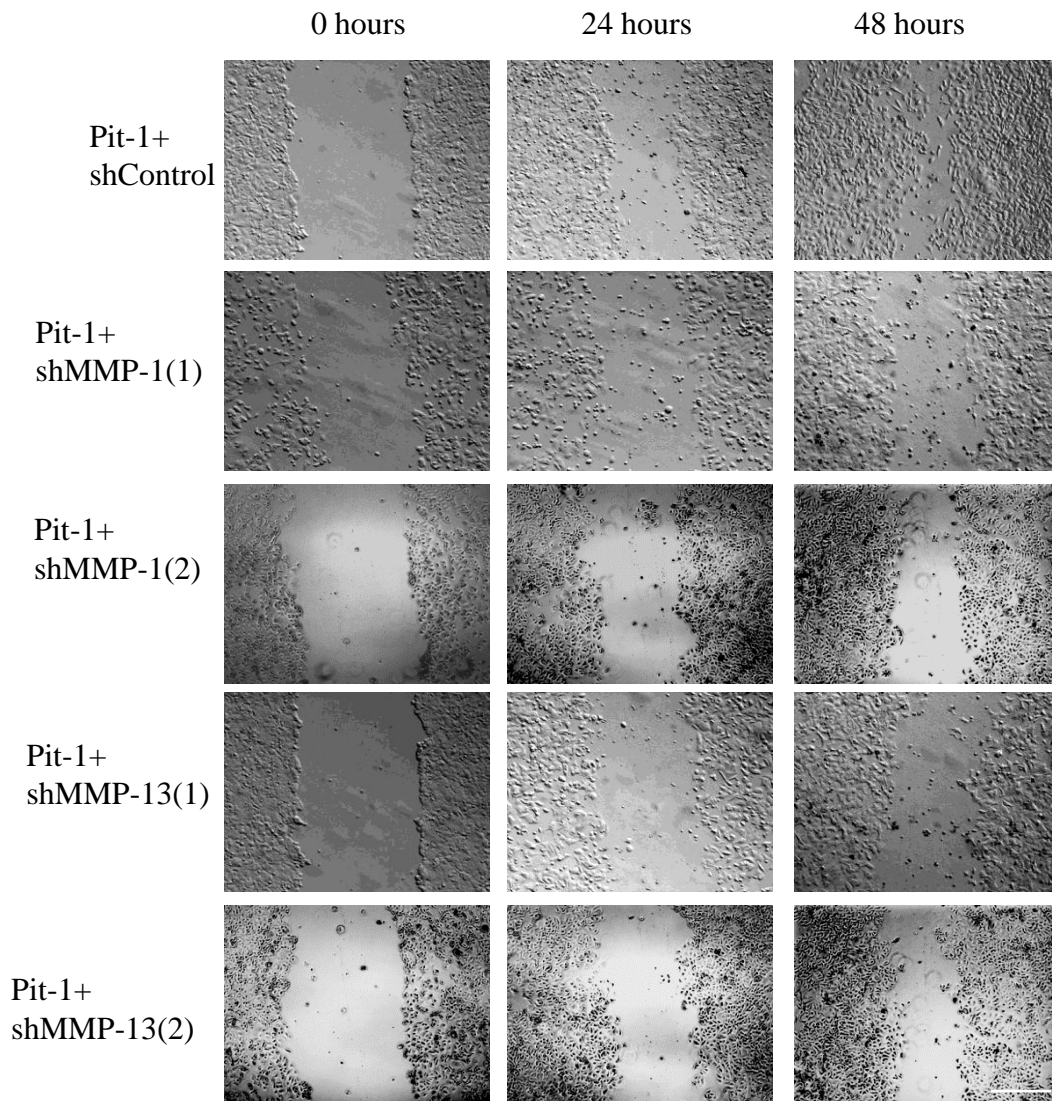

Supplement: Supplementary file 4 — Additional file 4: Figure S2.: Wound healing was carried out in MCF-7 cells with Pit-1 overexpression (pRSV-hPit-1), and knockdown of MMP-1 (shMMP-1(1) and shMMP-1(2)) and MMP-13 (shMMP-13(1) and shMMP-13(2)). Wounding was done using plastic pipette tip. At 24 and 48 hours, the distance between the wound edges was measured. Images were captured with an Olympus DP72 camera. Scale bar: 150 μm. (PDF 684 KB) [file 13058_2014_505_MOESM4_ESM.pdf]

MDA-MB-231 cells

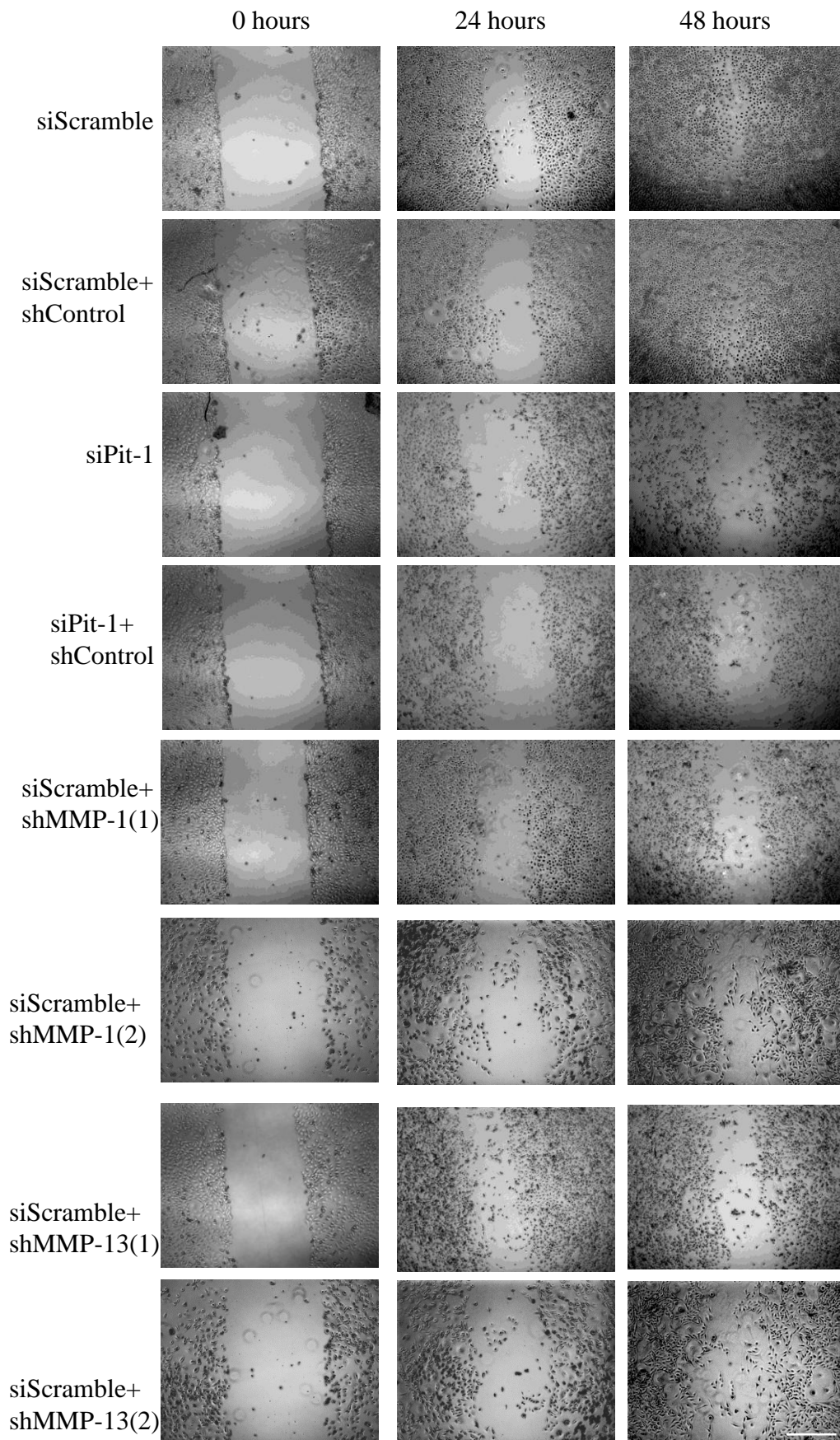

## MDA-MB-231 cells

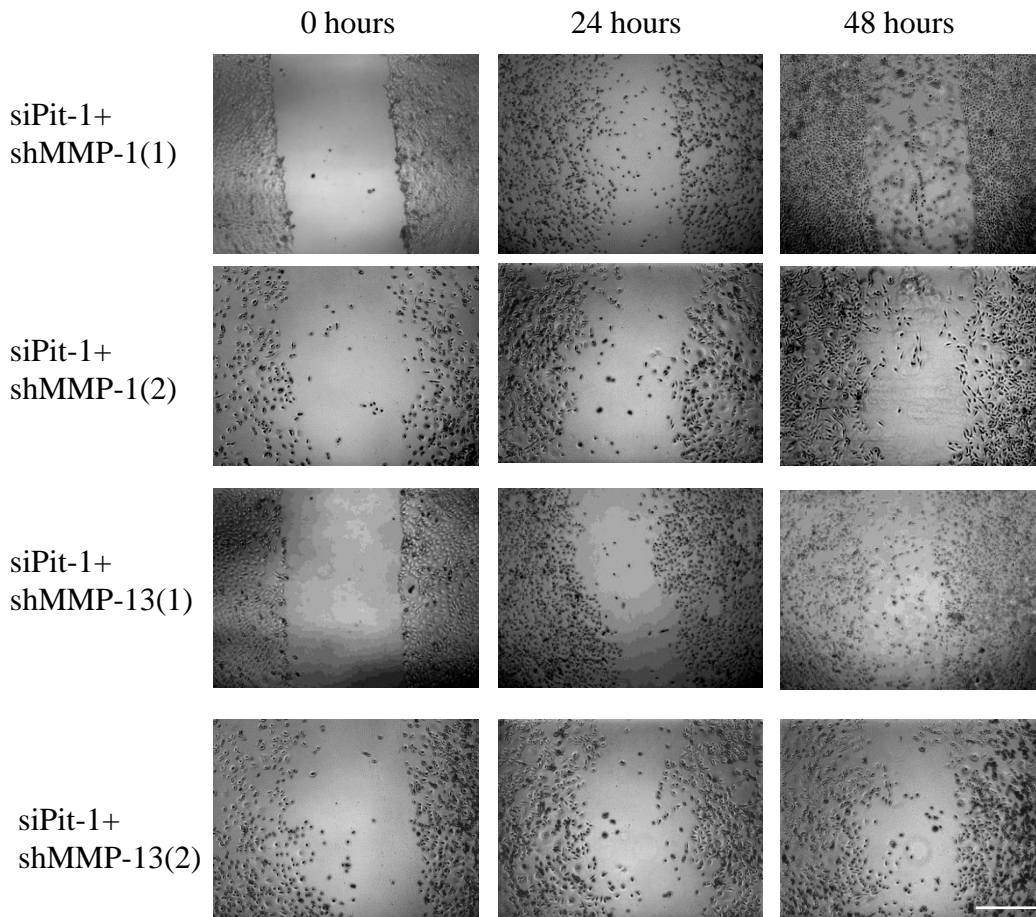

Supplement: Supplementary file 5 — Additional file 5: Figure S3.: Wound healing was carried out in MDA-MB-231 cells with Pit-1 knockdown (siPit-1), and knockdown of MMP-1 (shMMP-1(1) and shMMP-1(2)) and MMP-13 (shMMP-13(1) and shMMP-13(2)). Wounding was done using plastic pipette tip. At 24 and 48 hours, the distance between the wound edges was measured. Images were captured with an Olympus DP72 camera. Scale bar: 150 μm. (PDF 757 KB) [file 13058_2014_505_MOESM5_ESM.pdf]

A

Ki67 immunostaining

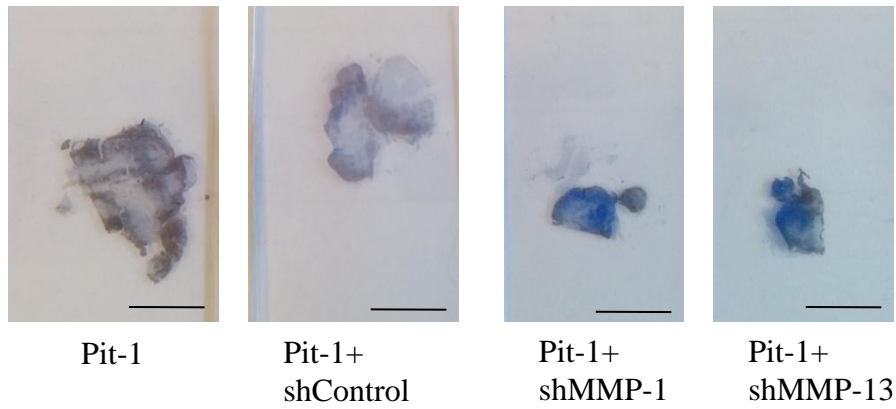

B

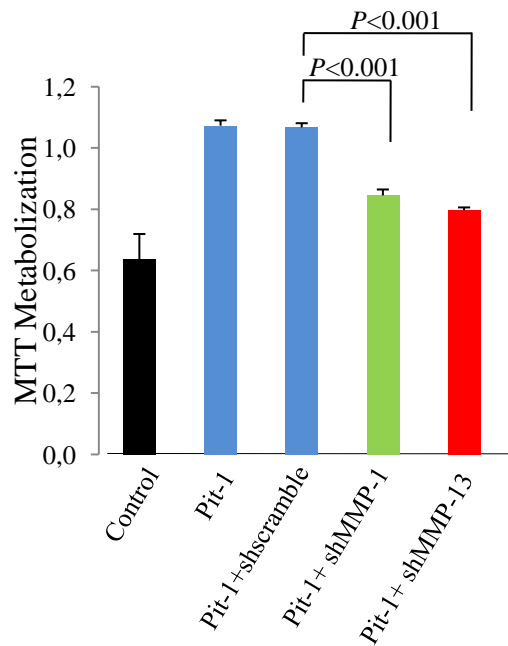

Supplement: Supplementary file 6 — Additional file 6: Figure S4.: MPP-1 and MMP-13 knockdown reduces tumor size. (A) Photos of slides showing tumors size at day 24 (in mice with Pit-1 overexpression, Pit-1, and Pit-1 + shControl) or day 33 (in mice injected with MCF-7-hPit-1-luc-shMMP-1 cells, or MCF-7-hPit-1- luc-shMMP-13 cells, Pit-1 + shMMP-1 and Pit-1shMMP-13, respectively), and immunostained with ki-67. Scale bar: 1 cm. (B) Cell proliferation (MTT) assay in MCF-7 control cells, MCF-7-hPit-1-luc cells, MCF-7-hPit-1-luc-shControl cells, MCF-7-hPit-1-luc-shMMP-1 cells, and MCF-7-hPit-1-luc-shMMP-13 cells. The absorbance of the samples was measured 48 h after transfection. Results were plotted as the mean ± SD values of quadruplicates from at least two independent experiments. (PDF 43 KB) [file 13058_2014_505_MOESM6_ESM.pdf]

**Pit-1 score values**

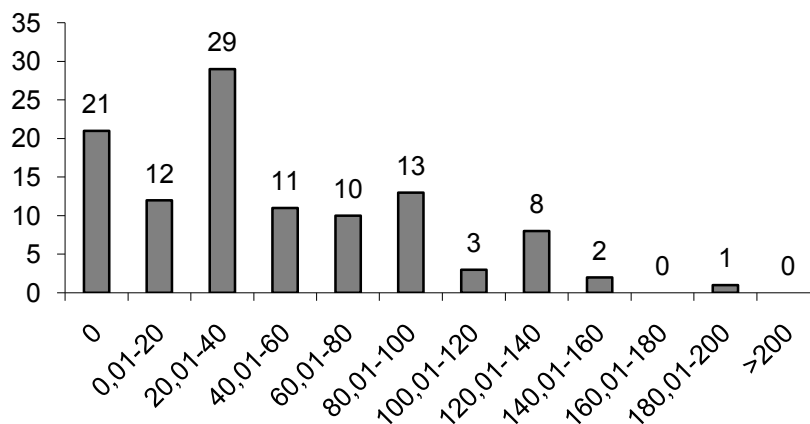

**MMP-1 score values**

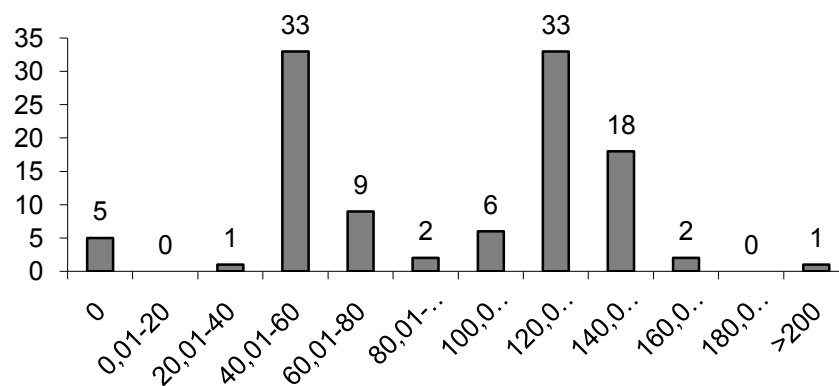

**MMP-13 score values**

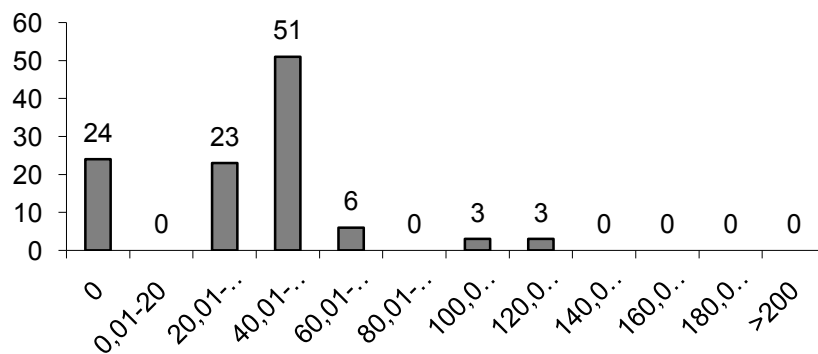

Supplement: Supplementary file 8 — Additional file 8: Figure S5.: Distribution score values obtained by immunohistochemical staining of Pit-1, MMP-1, and MMP-13 in 110 invasive ductal carcinomas of the breast. (PDF 232 KB) [file 13058_2014_505_MOESM8_ESM.pdf]

A

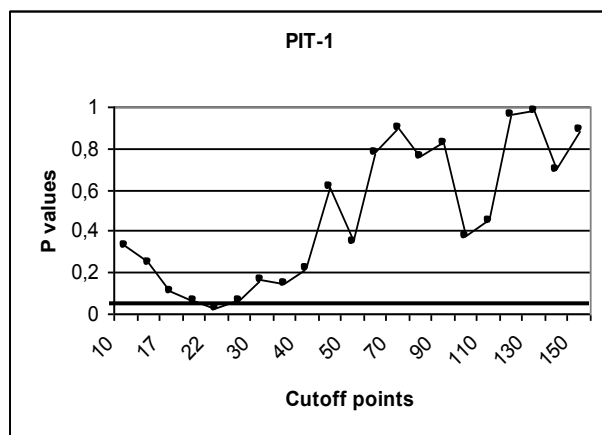

B

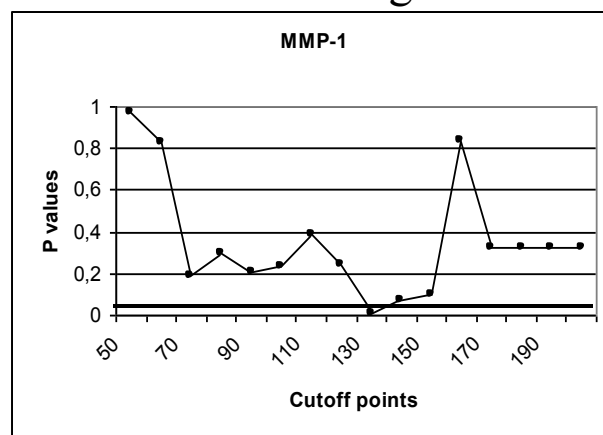

C

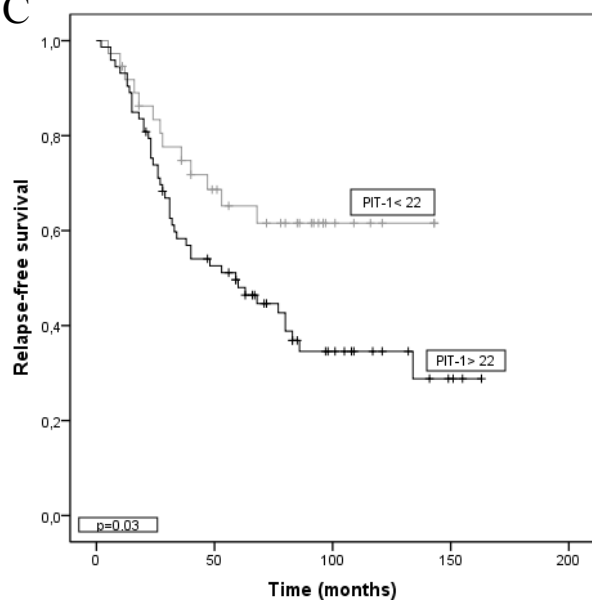

D

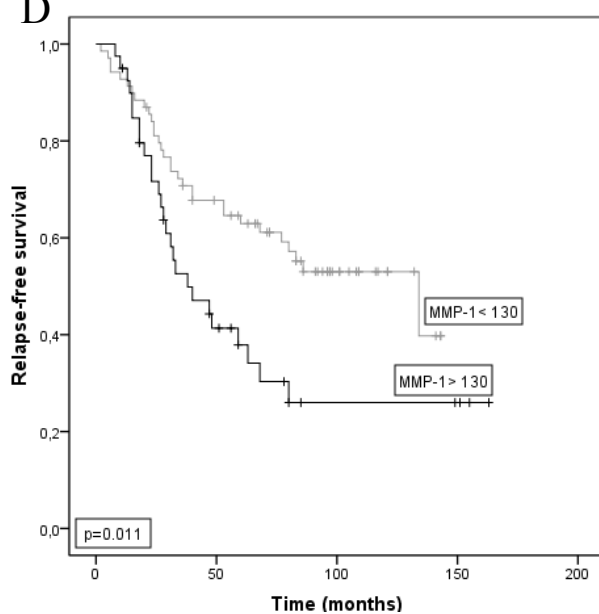

E

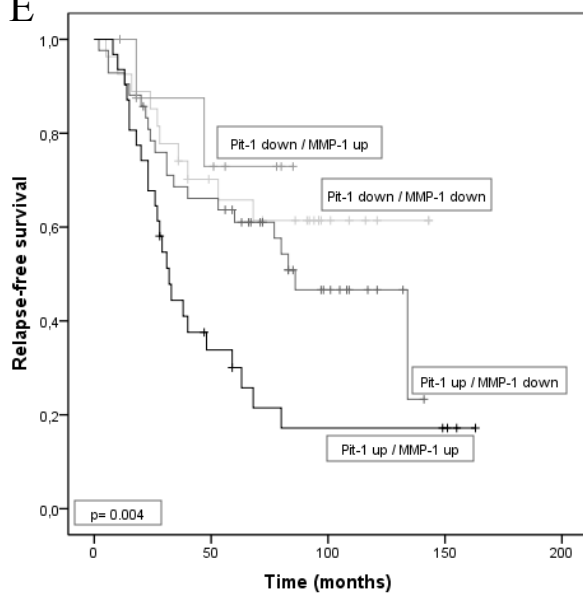

F

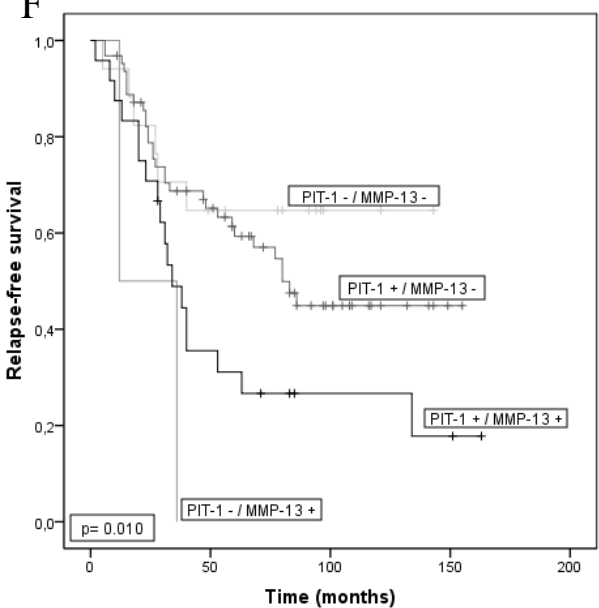

Supplement: Supplementary file 9 — Additional file 9: Figure S6.: Determination of cutoff values and their relationship with biochemical recurrence. Maximum likelihood determination of (A) Pit-1 and (B) MMP-1 cutoff values for predicting biochemical recurrence in 110 patients with breast cancer. The values obtained for each cutoff value are plotted against the value itself. Statistical significance is indicated by the horizontal line at 0.05. Analysis led to the definition of 22 for Pit-1 (χ 2 = 4.71, P = 0.03) and 130 for MMP-1 (χ 2 = 6.52, P = 0.011) as the optimal cutoff points. Probability of relapse-free survival as a function of the optimal cutoff point for (C) Pit-1 score values (P = 0.03), (D) MMP-1 (P = 0.011), and (E) the combination of both cutoff points (P = 0.004). (F) Probability of relapse-free survival as a function of Pit-1 and MMP-13 expression by inflammatory mononuclear cells. (PDF 210 KB) [file 13058_2014_505_MOESM9_ESM.pdf]

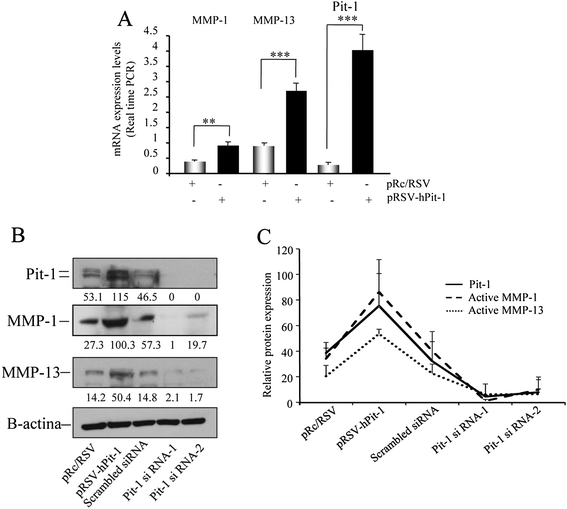

Supplement: Supplementary file 10 — Authors’ original file for figure 1 [file 13058_2014_505_MOESM10_ESM.gif]

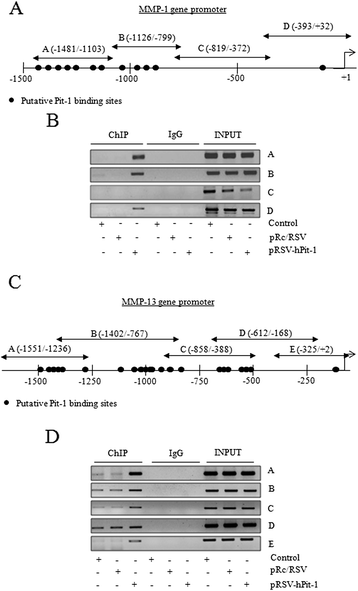

Supplement: Supplementary file 11 — Authors’ original file for figure 2 [file 13058_2014_505_MOESM11_ESM.gif]

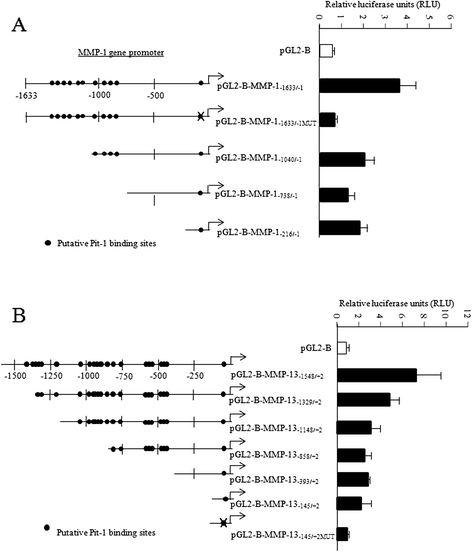

Supplement: Supplementary file 12 — Authors’ original file for figure 3 [file 13058_2014_505_MOESM12_ESM.gif]

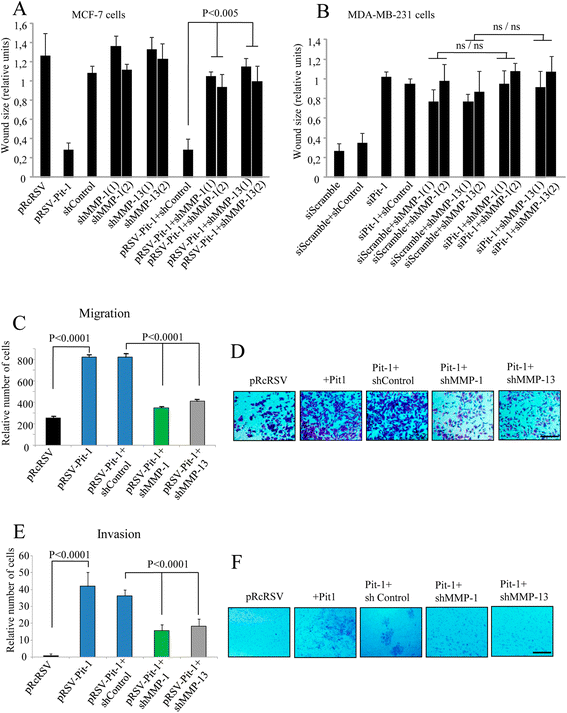

Supplement: Supplementary file 13 — Authors’ original file for figure 4 [file 13058_2014_505_MOESM13_ESM.gif]

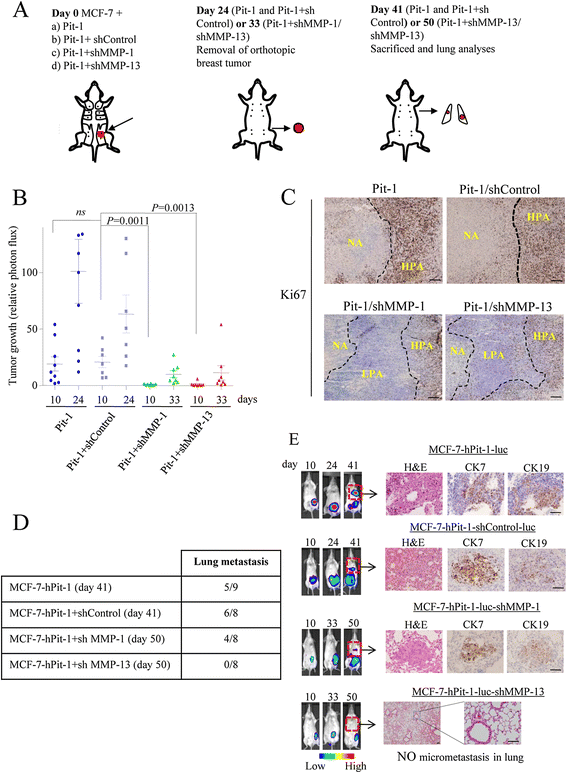

Supplement: Supplementary file 14 — Authors’ original file for figure 5 [file 13058_2014_505_MOESM14_ESM.gif]

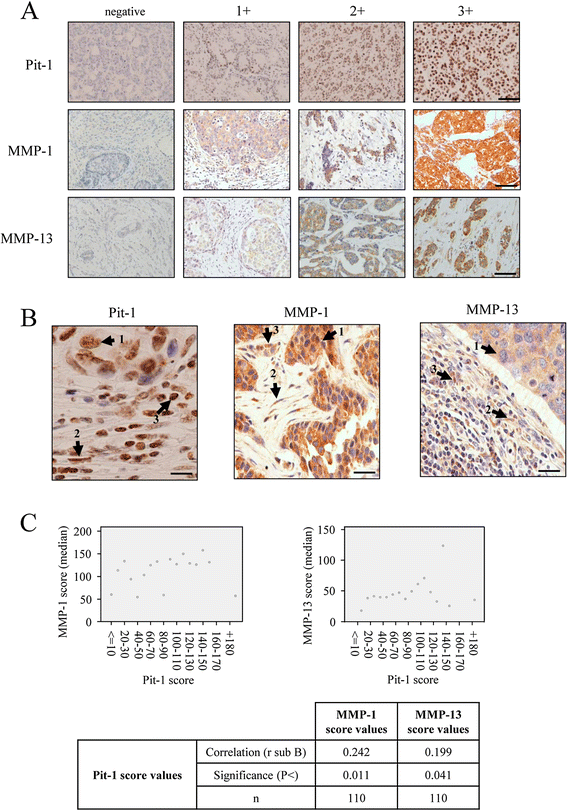

Supplement: Supplementary file 15 — Authors’ original file for figure 6 [file 13058_2014_505_MOESM15_ESM.gif]
