# Supplementary material for: Cancer progression by breast tumors with Pit-1-overexpression is blocked by inhibition of metalloproteinase (MMP)-13
Source: Breast Cancer Res. 2014 Dec 20;16:505. doi: 10.1186/s13058-014-0505-8 (PMC4305241; doi:10.1186/s13058-014-0505-8)
Supplement: Supplementary file 3 — Additional file 3: Table S1.: Basal characteristics of 110 patients with invasive ductal carcinoma of the breast. (PDF 74 KB) [file 13058_2014_505_MOESM3_ESM.pdf]

Additional file 3
Table S1

CHARACTERISTICS	Without recurrence No. (%)	With recurrence No. (%)
**Total cases**	52 (100)	58 (100)
**Age (years)**		
≤ 56.5	25 (48.1)	30 (51.7)
> 56.5	27 (51.9)	28 (48.3)
**Menopausal status**		
Premenopausal	17 (32.7)	17 (29.3)
Postmenopausal	35 (67.3)	41 (70.7)
**Tumoral size**		
T1	27 (51.9)	25 (43.1)
T2	25 (48.1)	33 (56.9)
**Nodal status**		
N (-)	27 (51.9)	25 (43.1)
N (+)	25 (48.1)	33 (56.9)
**Histological grade**		
Well Dif. (I)	19 (36.5)	13 (22.4)
Mod. Dif. (II)	27 (51.9)	26 (44.8)
Poorly Dif. (III)	6 (11.5)	19 (32.8)
**Nottingham prognostic index**		
<3.4	25 (48.1)	14 (24.1)
3.4-5.4	21 (40.4)	32 (55.2)
>5.4	6 (11.5)	12 (20.7)
**Estrogen Receptor**		
Negative	15 (28.8)	31 (53.4)
Positive	37 (71.2)	27 (46.6)
**Progesterone Receptor**		
Negative	19 (36.5)	39 (67.2)
Positive	33 (63.5)	19 (32.8)
**Adjuvant radiotherapy**		
No	40 (76.9)	32 (55.2)
Yes	12 (23.1)	26 (44.8)
**Adjuvant systemic therapy**		
Chemotherapy	14 (26.9)	25 (43.1)
Tamoxifen	21 (40.4)	14 (24.1)
Chemotherapy plus sequential Tamoxifen	10 (19.2)	6 (10.3)
No treatment	7 (13.5)	13 (22.4)
**HER2 Status**		
Negative	43 (82.7)	48 (82.8)
Positive	9 (17.3)	10 (17.2)
**Basal like phenotype**		
Non basal like	43 (82.7)	37 (63.8)
Basal like	9 (17.3)	21 (36.2)
