# Supplementary material for: Cancer progression by breast tumors with Pit-1-overexpression is blocked by inhibition of metalloproteinase (MMP)-13
Source: Breast Cancer Res. 2014 Dec 20;16:505. doi: 10.1186/s13058-014-0505-8 (PMC4305241; doi:10.1186/s13058-014-0505-8)
Supplement: Supplementary file 7 — Additional file 7: Table S2.: MMP-13 but not MMP-1 knockdown blocks metastasis in lung. (A) Number of metastasis in lung at day 41 (in MCF-7-hPit-1-luc- and MCF-7-hPit-1-luc-shControl-injected mice) or day 50 (in MCF-7-hPit-1-luc-shMMP-1-injected mice). Number was quantified after CK7 staining. Size represents mean + SD of total metastasis measurements. (B) Size (diameter in μm of each metastasis) and number of metastasis (specified for each mice) was evaluated using the Olympus DP-Soft morphometry program in an OlympusDX51 microscope. (PDF 186 KB) [file 13058_2014_505_MOESM7_ESM.pdf]

Additional file 7
Table S2

A

	Metastasis foci per lung						Size (X±SD)
Mice	1	2	3	4	5		
MCF-7-hPit-1-luc	2	6	38	15	13		166.2±174.1 μm
Mice	1	2	3	4	5	6	
MCF-7-hPit-1-luc-shControl	2	1	17	11	12	7	183.7+79.2 μm
Mice	1	2	3	4			
MCF-7-hPit-1-luc-shMMP-1	1	11	3	6			185.5±132.1 μm
Mice							
MCF-7-hPit-1-luc-shMMP-13		No	Foci				(-)

B

Metastasis size (diameter in μm):

**MCF-7-hPit-1-luc** Mice					**MCF-7-hPit-1-luc-shControl** Mice						**MCF-7-hPit-1-luc-shMMP-1** Mice			
1	2	3	4	5	1	2	3	4	5	6	1	2	3	4
58	45	71	770	342	281	52	151	242	163	74	99	159	339	357
1320	93	69	117	162	100		94	159	145	99		246	613	155
	56	205	42	244			169	170	98	231		267	232	102
	208	296	40	107			120	152	134	129		152		228
	100	78	61	131			246	359	153	77		102		60
	55	106	130	111			295	329	94	159		112		63
		160	101	132			296	135	221	211		170		
		93	72	84			123	150	196			77		
		109	97	88			290	144	148			218		
		114	68	116			263	129	213			60		
		181	186	168			330	105	188			86		
		177	56	104			220		165					
		76	84	75			229							
		192	95				148							
		234	108				356							
		177					131							
		175					321							
		153												
		148												
		357												
		215												
		93												
		179												
		80												
		118												
		262												
		352												
		241												
		94												
		435												
		160												
		164												
		157												
		138												
		186												
		264												
		109												
		60												
